# Bond-Transfer-Controlled Crack-Width Modelling of Reinforced Concrete as a Cement-Based Composite Under Imposed Strains

**DOI:** 10.3390/ma19194132

**Published:** 2026-09-28

**Authors:** Mariusz Zych, Marek Słoński

**Affiliations:** Faculty of Civil Engineering, Cracow University of Technology, 31-155 Cracow, Poland; marek.slonski@pk.edu.pl

**Keywords:** reinforced concrete, cement-based composite, crack width, crack spacing, imposed strains, reinforcement–concrete bond, bond transfer, end restraint, Eurocode 2, machine learning

## Abstract

**Highlights:**

• Reinforced concrete under imposed strains was analysed as a bond-controlled cement-based composite.
• A database of 137 specimens and 1473 measurement-level observations was compiled.
• EN 1992-1-1:2023 and MC2020 showed a stress-dependent underestimation of crack width.
• Bond- and stage-sensitive terms improved mean crack-width prediction from *R*^2^ = 0.72 to *R*^2^ = 0.83.
• Machine learning confirmed the dominant role of imposed strain and supported the stress-estimation model.

**Abstract:**

Reinforced concrete subjected to restrained imposed strains exhibits a cracking response governed by the interaction between concrete tensile behaviour, reinforcement–concrete bond transfer, crack-spacing development, reinforcement stress, and internal force redistribution. This study investigates this bond-controlled response at the material-system level and uses it to assess and extend the EN 1992-1-1:2023 crack-width formulation. A measurement-level database was compiled from published tests, comprising 137 specimens and 1473 filtered observations corresponding to successive imposed-strain or loading stages. The original EN 1992-1-1:2023 model reproduced the general trend but exhibited a systematic, stress-dependent underestimation, especially in the low-to-intermediate reinforcement stress range. To account for this range-dependent behaviour, bond- and stage-sensitive terms were introduced into the crack-spacing and mean strain-difference components. Within the calibration database, the modified model improved the agreement with measured mean crack widths from an *R*^2^ = 0.72 to *R*^2^ = 0.83 and reduced the underestimation rate relative to the estimated 95th-percentile crack width to 3.4% when measured reinforcement stress was used. Machine-learning analyses were used as diagnostic tools rather than replacements for the mechanics-based model. Gradient Boosting predicted mean crack width directly from imposed strain and material and geometrical parameters with *R*^2^ = 0.914, while SHAP interpretation confirmed the dominant role of imposed strain and the secondary influence of reinforcement ratio, bar diameter, and concrete tensile strength. These findings supported a semi-empirical stress-estimation relationship and a complete imposed-strain-based procedure. Within the calibration domain, the final procedure provides a mechanically interpretable, code-compatible estimate of upper-tail crack width that is equal to or greater than the adopted *w*_0.95_ reference for most measurement-stage observations.

## 1. Introduction

Reinforced concrete (RC) subjected to imposed deformations may be regarded as a cement-based composite in which cracking is governed by the coupled response of concrete in tension, the reinforcement–concrete interface, bond transfer, and post-cracking redistribution of internal forces. Cracking of RC members induced by imposed deformations is addressed less frequently in the literature than cracking under direct loading and is more difficult to model computationally, as discussed by Ng and Kwan [1], Carino [2], and Kaklauskas et al. [3]. For direct loads, such as pure bending or axial tension, the topic has been widely investigated in experimental studies by Gilbert and Nejadi [4] and Base [5], in theoretical studies by Pérez Caldentey et al. [6] and Borosnyói and Balázs [7], and in assessments and background documents for design provisions [8,9]. Related evidence from fibre-reinforced cementitious composites also demonstrates the importance of interface-controlled load transfer for crack development. Bai et al. [10] showed that modification of the PE fibre–matrix interface in recycled-powder engineered cementitious composites affected tensile behaviour and promoted finer, more distributed cracking. Although fibre bridging in ECC differs from the reinforcement–concrete bond mechanism considered in the present study, these results further illustrate the broader sensitivity of cracking in cement-based composites to interfacial properties.

Although successive European codes have introduced relationships for crack-width calculation under imposed strains (CEN [11], CEN [12], CEN [13]), their ability to represent the underlying bond-controlled cracking mechanism remains only partly verified against experimental data. The validity and accuracy of engineering models used in design practice can be established only by comparison with experimental results. The need for experimentally grounded crack-control assessment has also been emphasised in the CEOS.fr research programme on reinforced concrete cracking [14]. For members subjected to imposed strains and restrained at both ends, the earlier codes (CEN [11], CEN [12]) were based on Beeby’s [15] theoretical approach. In contrast, the most recent version (CEN [13]) adopts a different strategy: for such restrained members, the recommended relationship is calibrated using results obtained under direct axial loading.

It therefore remains debatable whether the loading type, either direct or imposed, should determine the calculation of the mean strain difference between reinforcement and concrete. Although crack-width development is associated in both cases with reinforcement slip relative to concrete under increasing axial force, the actual force and displacement fields may differ substantially depending on restraint conditions, as indicated by Kaklauskas et al. [3]. The simplifying assumption adopted in CEN [13], which equates the effects of direct axial tension with those of imposed strains, has not yet been supported by sufficient experimental validation.

This study does not address members restrained along their base, which were examined in detail by Aghaee et al. [16]. Instead, it focuses on members restrained at both ends. The main novelty is an integrated treatment of reinforcement stress, bond-transfer behaviour, and cracking-stage development within the general structure of the CEN [13] crack-width formulation. Unlike existing code-based approaches, in which reinforcement stress is prescribed as an input and the bond- and tension-stiffening-related parameters remain constant, the proposed procedure (i) estimates reinforcement stress directly from imposed strain and selected material and reinforcement parameters; (ii) introduces a stress-dependent bond-related correction into the crack-spacing term; and (iii) adjusts the mean strain-difference term using the ratio *σ_cr_*/*σ_s_* as a practical indicator of cracking development. These components are calibrated and assessed using measurement-level data from successive deformation stages rather than only a single cracking point. Machine learning is used to identify and verify the governing variables, while the final prediction procedure remains mechanically interpretable and compatible with the Eurocode format. Thus, the principal contribution is the combination of these elements into a complete imposed-strain-based crack-width calculation procedure for end-restrained members.

## 2. Mechanistic Background and Research Gap

### 2.1. Restraint-Induced Cracking, Bond Transfer and Crack Formation Stages

In reinforced concrete considered as a cement-based composite with discrete steel reinforcement and a bond-governed interface, cracking under imposed strains differs fundamentally from cracking under direct mechanical loading. In load-controlled members, the internal force and reinforcement stress follow directly from equilibrium with the applied load. In end-restrained members subjected to imposed strain, the internal force develops through compatibility with the restraints, crack formation, bond transfer, and stress redistribution. Imposed strain, reinforcement stress, and crack width are therefore coupled response quantities rather than independently prescribed variables.

This distinction has been emphasised in studies on restrained and shrinkage-induced cracking. Carino [2] showed that restrained cracking depends not only on tensile strength, but also on restraint conditions, time-dependent deformation and post-cracking stress redistribution. Ng and Kwan [1] demonstrated that shrinkage cracking requires a compatibility-based analytical treatment, while Kaklauskas et al. [3] showed that shrinkage affects tension stiffening and the mean deformation response of reinforced concrete members.

For members restrained at opposite ends, cracking develops progressively as the imposed strain increases. In the early cracking range, only one or a few cracks may form, and the effective crack spacing may be much larger than the spacing associated with stabilised cracking. Bond transfer then develops over longer transfer lengths, and the stress-transfer mechanism differs from the stabilised pattern assumed in many design formulations. As the imposed strain increases further, additional cracks form, crack spacing decreases, and the response gradually approaches a more developed cracking state.

The experimental basis for this specific problem is much narrower than for members under direct loading. The tests by Farra and Jaccoud [17] remain among the key sources for RC ties subjected to short-term imposed deformation because they provide imposed strain, reinforcement stress, and crack-width measurements at successive deformation stages. Further evidence is provided by Rimkus and Gribniak [18], who investigated concrete ties reinforced with multiple bars, and by García and Pérez Caldentey [19], who analysed the influence of loading type on cracking behaviour. These studies show that reinforcement layout and loading scheme may substantially affect crack spacing, mean deformations, and crack-width development.

From a material-system point of view, crack-width development is governed by the interaction between concrete tensile cracking, reinforcement–concrete bond transfer, relative slip, and tension stiffening. After cracking, tensile force is transferred from steel to concrete by bond stresses, whose magnitude and distribution depend on concrete tensile properties, bar diameter, reinforcement ratio, crack spacing, relative slip and reinforcement stress. The importance of bond and crack spacing has also been emphasised in crack-control models. Borosnyói and Balázs [7] summarised the role of crack spacing, reinforcement arrangement and bond assumptions in flexural cracking models, while Pérez Caldentey et al. [6] showed that cover, the bar diameter-to-effective reinforcement ratio and reinforcement detailing influence crack spacing and crack width.

Thus, the same restrained member may pass from a few-crack state to a more developed cracking pattern during a single imposed-deformation history. This stage dependence provides the mechanical basis for re-examining whether a constant bond-related term in the EC2023 crack-spacing expression can represent the full experimental response.

The present study focuses on members restrained at opposite ends and subjected to imposed strains, or on loading conditions from which the imposed strain can be reconstructed.

### 2.2. Eurocode Crack-Width Models for Imposed Strains and Their Limitations

European crack-width models have evolved from EN 1992-1-1:2004 [11] and EN 1992-2:2006 [12] towards the second-generation formulation in EN 1992-1-1:2023 [13]. In all these approaches, the calculated crack width is generally obtained as the product of a crack-spacing term and a mean strain-difference term. Both components include simplified assumptions concerning bond, tension stiffening, and cracking stage.

For members restrained at both ends and subjected to imposed strain, the earlier Eurocode provisions were closely related to Beeby’s theoretical approach [11,12,15]. The current EN 1992-1-1:2023 formulation adopts a different strategy, in which the procedure for restrained members is linked to assumptions calibrated mainly using results from direct axial loading [13]. This raises a fundamental question: can direct axial tension and imposed deformation be treated as mechanically equivalent for crack-width calculation? Although crack opening is associated in both cases with reinforcement slip relative to concrete, reinforcement stress may differ because of restraint conditions, cracking sequence, bond transfer, and internal force redistribution.

A key limitation of the standardised procedure, when applied to imposed-strain cracking, is that reinforcement stress is treated as an input although it is in fact a response variable governed by compatibility, bond transfer, crack sequence, and internal force redistribution. Under direct loading, *σ_s_* can be obtained from the applied load and section equilibrium. In end-restrained members subjected to imposed strain, *σ_s_* is part of the structural response and evolves with the cracking sequence, bond transfer, and internal force redistribution. Consequently, the EC2023 procedure requires a stress input that cannot be specified independently of the imposed deformation for the member type considered here.

Another difficulty is that the strain-difference and crack-spacing terms depend on the cracking stage assumed in the calculation. Eurocode-type formulations generally rely on parameters calibrated for developed cracking conditions, whereas the stress level adopted for restrained imposed-strain cases may correspond to an earlier cracking range. As noted by Schlicke et al. [20], this stress level does not necessarily ensure stabilised crack spacing. This creates a potential inconsistency between the stress input and the spacing assumptions embedded in the EC2023 formulation. Recent studies on Eurocode, fib Model Code, and modified tension chord approaches confirm that standardised models for imposed-strain cracking require careful interpretation and calibration [21,22,23,24].

Experimental results by Farra and Jaccoud [17] support this concern because they document successive imposed-deformation stages with evolving reinforcement stress, crack width, and number of cracks. These observations confirm that cracking response should be evaluated over the full experimental deformation history, not only at the theoretical cracking-force level. This progressive behaviour is also consistent with the need to consider member relaxation after successive cracking events, as discussed by Zych [25].

Consequently, the applicability of EN 1992-1-1:2023 to end-restrained members subjected to imposed strain should be assessed over the experimentally observed range of imposed strains, reinforcement stresses, and crack widths, rather than only at the theoretical cracking-force level. This also motivates the development of a supplementary *σ_s_*(*ε_imp_*) relationship compatible with the EC2023 crack-width procedure.

### 2.3. Research Gaps and ML-Supported Interpretation

Model assessment should distinguish between prediction of the mean cracking response and evaluation relative to upper-tail crack-width measures. Comparisons with measured mean crack widths indicate whether the model captures the average mechanical response, whereas comparisons with maximum or upper-tail crack-width measures are needed because excessive crack openings are not acceptable in SLS. This interpretation is consistent with probabilistic approaches to crack-width assessment [26,27].

Machine learning is used as a diagnostic and interpretative tool that also supports the selection of candidate variables for the subsequent semi-empirical model. It is not used to generate or replace the final engineering formulation directly. Applications of machine learning in concrete technology have increasingly included both the prediction of crack-width-related response quantities and image-based detection and assessment of crack development [28,29,30]. In the present context, ML serves two purposes. First, it tests whether the mean crack width can be predicted directly from imposed strain and material-geometrical parameters, without measured reinforcement stress as an input. Second, it supports the identification of variables governing the transformation from imposed strain to reinforcement stress, which is later used to formulate a semi-empirical *σ_s_*(*ε_imp_*) relationship. Interpretable techniques such as SHAP [31] are therefore used to verify whether statistically important variables are also mechanically meaningful.

A relevant methodological precedent was provided by Aghaee et al. [16], who used ensemble ML models to predict restraint-induced crack widths in externally restrained RC structures. Their RF and XGB models outperformed standardised approaches including Eurocode 2, CIRIA C766 [32], ACI 207 [33], and ACI 224 [34], while feature-importance and SHAP analyses indicated that the predictions were governed by physically meaningful variables. Although that study focused mainly on edge-restrained wall-type members, this precedent supports the use of ML as a diagnostic tool for identifying governing variables and assisting the calibration of simplified engineering formulations.

The preceding review identifies three main gaps:

• First, the bond-controlled cracking response of end-restrained reinforced concrete members subjected to imposed strains has not been systematically assessed at the measurement level.
• Second, the reinforcement stress required in the EC2023 crack-width calculation procedure is not an independently prescribed quantity, but a response variable governed by imposed strain, bond transfer, crack sequence, and internal force redistribution.
• Third, the bond and crack-spacing assumptions embedded in standardised crack-width models may be insufficient to represent the transition from early few-crack stages to more developed cracking patterns.

The present study addresses these gaps by compiling a measurement-level experimental database and by analysing how bond transfer, reinforcement stress, and cracking stage affect the performance of the EC2023 crack-width formulation over the experimentally observed response range. Based on this assessment, a bond-sensitive modification of the crack-spacing term is introduced, together with a semi-empirical relationship for estimating reinforcement stress from imposed strain and selected material and reinforcement parameters.

## 3. Experimental Database and Evaluation Methodology

### 3.1. Source Studies and Selection Criteria

The experimental database used in this study was compiled from the test results reported by Farra and Jaccoud [17], Rimkus and Gribniak [18], and García and Pérez Caldentey [19]. The source studies were selected using two main criteria. First, the tests had to concern reinforced concrete members subjected to imposed deformation or loading conditions for which the corresponding imposed strain could be reconstructed with sufficient reliability. Second, the reports had to provide the information required for an unambiguous application of code-based crack-width models, including material properties, reinforcement layout, imposed strain or load level, reinforcement stress, and measured crack widths. Studies with incomplete data were not included because supplementing missing parameters by general assumptions would introduce additional uncertainty and could obscure the comparison between calculated and measured crack widths.

### 3.2. Measurement-Level Database and Filtering Procedure

In total, 137 experimental cases were identified: 121 from Farra and Jaccoud [17], 10 from Rimkus and Gribniak [18], and six from García and Pérez Caldentey [19]. Because many specimens, particularly those tested by Farra and Jaccoud [17], were monitored at successive loading or imposed-deformation stages, two data levels were distinguished. The specimen-level database comprised the 137 experimental cases, whereas the measurement-level database treated each imposed-strain or loading stage as a separate observation. This measurement-level format was adopted because it enables crack-width evolution to be analysed over successive deformation stages.

The database was intentionally restricted to reinforced-concrete members restrained at opposite ends and subjected to longitudinal imposed strain, because this structural configuration corresponds to the cracking mechanism and code-based formulations examined in the subsequent analyses. Although the Farra and Jaccoud [17] programme accounts for most specimens, it covers the widest range of concrete material properties among the three source programmes. This concentration reflects the scarcity of fully documented experimental studies on end-restrained members under imposed strains in existing literature. To mitigate the risk of dataset-specific bias and evaluate model transferability, group-based train/test partitioning was strictly enforced at the specimen level, ensuring that the model’s accuracy is verified on unseen structural configurations. Furthermore, input features were defined as normalised mechanical and geometric parameters. While SHAP analyses confirm that the models capture physically consistent relationships across the dataset, the proposed semi-empirical and ML models should be applied within the calibrated domain. Further experimental validation on independent testing programmes with diverse geometries and boundary conditions remains a valuable direction for future research.

Before the code-based and machine-learning analyses, a quality-control and consistency assessment was performed. Completeness of the variables required for the application of the crack-width models had already been assessed during selection of the source experimental programmes. Only programmes providing all required variables were included; therefore, no missing values were imputed, and all 137 eligible specimens were retained.

The subsequent filtering was performed at the measurement-stage level. Because the database describes progressive deformation histories, physically based criteria were applied rather than statistical outlier removal. The criteria were applied sequentially in the following order: (1) unloading stages and observations showing no increase in reinforcement stress relative to the preceding stage were excluded; (2) among the remaining data, observations with *w_max_* < 0.03 mm were excluded because such small openings may be affected by measurement uncertainty, initial microcracking, or difficulties in identifying newly formed cracks; and (3) observations with *σ_s_* > 500 MPa were excluded because reinforcement yielding may result in crack-width development that is no longer representative of serviceability conditions. An observation satisfying more than one criterion was assigned only to the first applicable exclusion category. Therefore, the exclusion counts in Table 1 depend on the stated order of screening and do not represent independent totals for the respective criteria. The filtering sequence reduced the initial 3099 measurement-stage observations to a final database of 1473 observations.

The distributions of the main variables are shown in Figure 1. The dataset covers a relatively broad but non-uniform experimental domain, with most observations corresponding to normal-strength concrete, low-to-moderate imposed strain levels, and serviceability-range crack widths. The reinforcement stress range extends from low stress levels up to approximately 500 MPa, allowing the EC2023 model to be assessed over the experimentally observed response range rather than only near the theoretical cracking-force level. Because several observations originate from successive stages of the same specimens, the database should be interpreted as a measurement-level dataset, not as a statistically independent specimen-level population.

The effective reinforcement ratio, (*ρ_eff_*), used throughout this study was determined using the formulations provided in EC2023.

### 3.3. Statistical Overview of the Experimental Database

Table 2 provides a statistical summary of the filtered measurement-level database used in the subsequent code-based and machine-learning analyses. The reported ranges define the experimental domain within which the analysed models were assessed and calibrated. The differences between mean and median values, especially for imposed strain and crack-width-related quantities, confirm the non-uniform structure of the dataset already shown in Figure 1.

To further characterise the internal structure of the database, Pearson correlation coefficients were calculated for the main geometrical, material, reinforcement-related, and response variables (Figure 2). The correlation map shows strong positive associations between imposed strain *ε_imp_* and the measured crack-width quantities. The correlation coefficients between *ε_imp_* and *w_m_* and between *ε_imp_* and *w_max_* are *r* = 0.78 and *r* = 0.83, respectively. Reinforcement stress shows even stronger correlations with crack width, with *r* = 0.90 for *σ_s_*-*w_m_* and *r* = 0.86 for *σ_s_*-*w_max_*. These relationships confirm that both imposed strain and reinforcement stress are strongly linked with crack-width development in the analysed database.

Negative correlations are observed between crack width and reinforcement-related parameters, particularly reinforcement ratio and bar diameter. These trends are mechanically consistent with the crack-width-limiting effect of reinforcement, but they should be interpreted with caution because several input variables are mutually dependent. In particular, *ϕ* and *ρ* are strongly correlated in the present database, while other dependencies, such as those between *n* and *A_ct_*, reflect the limited number of reinforcement layouts and specimen geometries available in the source studies.

Overall, the Pearson correlation matrix provides a useful preliminary view of the database structure and supports the subsequent selection of variables for code-based assessment and ML interpretation. Because successive observations from the same specimen are correlated, the reported coefficients represent descriptive associations within the pooled measurement-level database, combining within-specimen response evolution with between-specimen differences, rather than inferential statistics based on 1473 independent observations. Specimens represented by a larger number of measurement stages may also have a greater influence on these coefficients. Furthermore, the correlations should not be interpreted as isolated parametric effects, because the source studies include a limited number of geometries and reinforcement layouts.

### 3.4. Reference Crack-Width Measures and Reliability Interpretation

The evaluation of crack-width models requires a clear definition of the crack-width measure used for comparison. In EN 1992-1-1, the calculated crack width *w_k_* should be interpreted as a design-oriented serviceability value rather than as a direct estimator of either the mean or the absolute maximum crack width [11]. This interpretation is consistent with Eurocode background documents and standard reinforced-concrete design references [8,35,36,37].

In the present study, comparisons with the measured mean crack width *w_m_* are used to assess whether the model captures the average mechanical response. Comparisons with the approximated or empirical 95th-percentile crack width, *w*_0.95,_*_pprox._* or *w*_0.95,_*_emp_*, respectively, and the measured maximum crack width *w_max_* are used to evaluate the upper-tail response. This distinction is consistent with serviceability-oriented and probabilistic interpretations of crack-width assessment [26,27].

When the full distribution of crack widths within a specimen was not available, the 95th-percentile crack width was approximated from the measured mean and maximum crack widths. As a simplified engineering approximation, the maximum measured crack width was assumed to correspond to approximately three standard deviations above the mean:

(1) $$w_{max}\approx \bar{w}+3s$$

where $\bar{w}$ is the mean crack width, *w_max_* is the maximum observed crack width, and *s* is the standard deviation of crack widths. The standard deviation can then be estimated as:

(2) $$s\approx \left(w_{max}-\bar{w}\right)/3$$

The approximated 95th-percentile crack width was then calculated as:

(3) $$w_{0.95,approx}\approx \bar{w}+1.645s$$

This approximation should be regarded as a practical estimate because its accuracy depends on the actual shape of the crack-width distribution. Nevertheless, simplified statistical interpretations of crack-width data are commonly used when only limited information is available [26,27,35].

In the following, values obtained from Equations (1)–(3) are denoted by *w*_0.95,_*_pprox._*. Where complete crack-width distributions were available, the empirical 95th percentile, denoted by *w*_0.95,_*_emp_*, was calculated directly using linear percentile interpolation.

The approximation was evaluated using the complete crack-width distributions available from Rimkus and Gribniak [18] and García and Pérez Caldentey [19]. The empirical 95th percentile, *w*_0.95,_*_emp_*, was determined directly from these distributions and compared with *w*_0.95,_*_pprox._* obtained from Equations (1)–(3). Overall, *w*_0.95,_*_pprox._* was approximately 7% lower than *w*_0.95,_*_emp_* on average and was lower in most comparisons. The approximation is therefore treated as a practical but moderately lower-biassed upper-tail estimate rather than an exact empirical percentile.

Throughout the model assessment, *w_ref_* denotes the crack-width quantity used as the experimental reference in a given comparison, namely *w_m_*, *w*_0.95_, or *w_max_*. The signed prediction error is defined as:

(4) $$e_{i}=w_{calc,i}-w_{ref,i}$$

Thus, *e_i_* < 0 denotes underestimation and *e_i_* > 0 denotes overestimation. The underestimation rate is the number of cases for which *e_i_* < 0, divided by the total number of cases and expressed as a percentage. Because these proportions are calculated at the measurement-stage level, they describe the frequency of underestimation across the observed deformation domain and should not be interpreted as proportions derived from statistically independent specimens. For underestimated cases, the relative underestimation is defined as:

(5) $$u_{rel,i}=\frac{w_{ref,i}-w_{calc,i}}{w_{ref,i}}100\%$$

### 3.5. Machine-Learning Methodology

The ML analysis was used as a diagnostic tool to examine whether imposed strain and selected material, geometrical, reinforcement, and bond-related variables contain sufficient information to reproduce the cracking response of end-restrained RC members. The ML methodology was divided into two analytical benchmarks targeting different dependent variables. Four regression models were selected to capture both linear trends and nonlinear interactions between variables: Linear Regression (LR), Random Forest (RF), Gradient Boosting (GB), and CatBoost (CB) [38]. LR provided a baseline, while the tree-based models were selected for their ability to represent nonlinear physical relationships without predefined functional forms. The selection of these model families is consistent with recent applications and reviews of ML in concrete-related prediction tasks [16,28,29].

The LR model was used as a baseline for mapping the relationship between the dependent variables, such as mean crack width or reinforcement stress, and multiple independent predictors under the assumption of linearity. Although LR can capture general global trends in the data, its predictive capability is limited when the response depends on the nonlinear interactions between imposed strain, reinforcement layout, and material properties. Furthermore, LR is sensitive to correlated variables, which may distort model coefficients and lead to unstable or misleading estimates of individual feature effects. These limitations explain why the purely linear approximation is expected to show a larger prediction scatter than nonlinear ensemble models.

RF is a nonlinear ensemble learning algorithm that aggregates multiple decision trees to generate predictions [39]. It relies on bootstrap aggregation, where individual trees are trained on random samples drawn with replacement from the original dataset. Additional randomness is introduced by considering only a random subset of features at each node split. The final prediction is obtained by averaging the outputs of all trees, which reduces prediction variance and improves model stability. RF is therefore useful for nonlinear regression tasks and provides a robust reference among tree-based models.

GB is a nonlinear supervised model based on ensemble learning [40]. Unlike RF, which trains independent trees in parallel, GB builds the model sequentially by combining weak learners, typically shallow decision trees. Each new learner is fitted to reduce the residual error of the previous model, which allows GB to capture complex relationships with high accuracy. However, this sequential bias-reduction mechanism also requires appropriate regularisation to reduce the risk of overfitting.

CB is a gradient-boosting algorithm that uses ordered boosting and symmetric decision trees to improve generalisation and reduce prediction bias [41]. In this study, it was included because tree-based boosting models are well suited to nonlinear interactions between imposed strain, reinforcement layout, concrete properties, and crack-width response, while still allowing post hoc interpretation using SHAP values.

To ensure reproducibility of the data-driven models, all machine-learning pipelines were developed in Python (ver. 3.10) using scikit-learn (ver. 1.8), catboost, and shap libraries under a fixed global random seed (seed = 43 and n_estimators = 100). Data partitioning into training (80%) and testing (20%) sets was conducted at the specimen level, guaranteeing that all sequential loading measurements from any individual specimen were placed exclusively in either the training or testing subset. Feature scaling (Z-score normalisation) was applied only for Linear Regression, whereas decision-tree algorithms (Random Forest, Gradient Boosting, CatBoost) were trained on unscaled features due to their native scale-invariance. The experiments were performed using Google Colab.

To account for the hierarchical structure of the database, the training–testing partition was performed at the specimen level. All measurement stages associated with a given specimen were assigned exclusively to either the training set (80%) or the testing set (20%). Thus, no observations from the same specimen occurred in both subsets, preventing information leakage between successive measurement stages. The reported testing metrics remain measurement-level indicators, but they are calculated exclusively for observations belonging to specimens not used during model training.

Because the experimental database includes 1473 measurement stages obtained from 137 distinct specimens, successive observations within the same test series exhibit intra-specimen correlation. While measurement-level data is essential for modelling the progressive evolution of crack width from initial to stabilised cracking stages, standard statistical assumptions of independence do not apply across rows originating from the same element. To ensure that this dependence does not artificially inflate reported machine-learning performance metrics, a group-based statistical treatment was implemented. Data partitioning was performed strictly at the specimen level, ensuring that all measurement stages of a given specimen were kept within the same fold. Consequently, the reported testing error metrics (*R*^2^, RMSE, MAE) evaluate performance exclusively on unseen specimen configurations, confirming the statistical robustness and generalizability of the proposed models. The reported testing metrics remain measurement-level indicators, but they are calculated exclusively for observations belonging to specimens not used during model training. This procedure is consistent with grouped validation strategies recommended for hierarchically structured data [42].

Each model’s predictive accuracy was assessed using a testing dataset and three standard metrics: *R*^2^, RMSE and MAE. *R*^2^ was used to quantify the proportion of variance explained by the model, whereas RMSE and MAE described the magnitude of prediction errors.

## 4. Baseline Assessment of the EC2023 and MC2020 Crack-Width Models

### 4.1. Prediction of Mean Crack Width

The mean crack widths calculated using the EC2023 [13] and fib Model Code 2020 [43] formulations were compared with the corresponding measured mean values (Figure 3a,b). For EC2023, the correction factor (*k_w_* = 1.7) was omitted to obtain mean values. The analysis covered the full range of reinforcement stress levels represented in the experimental database, i.e., approximately 50–500 Mpa.

**Figure 3:**
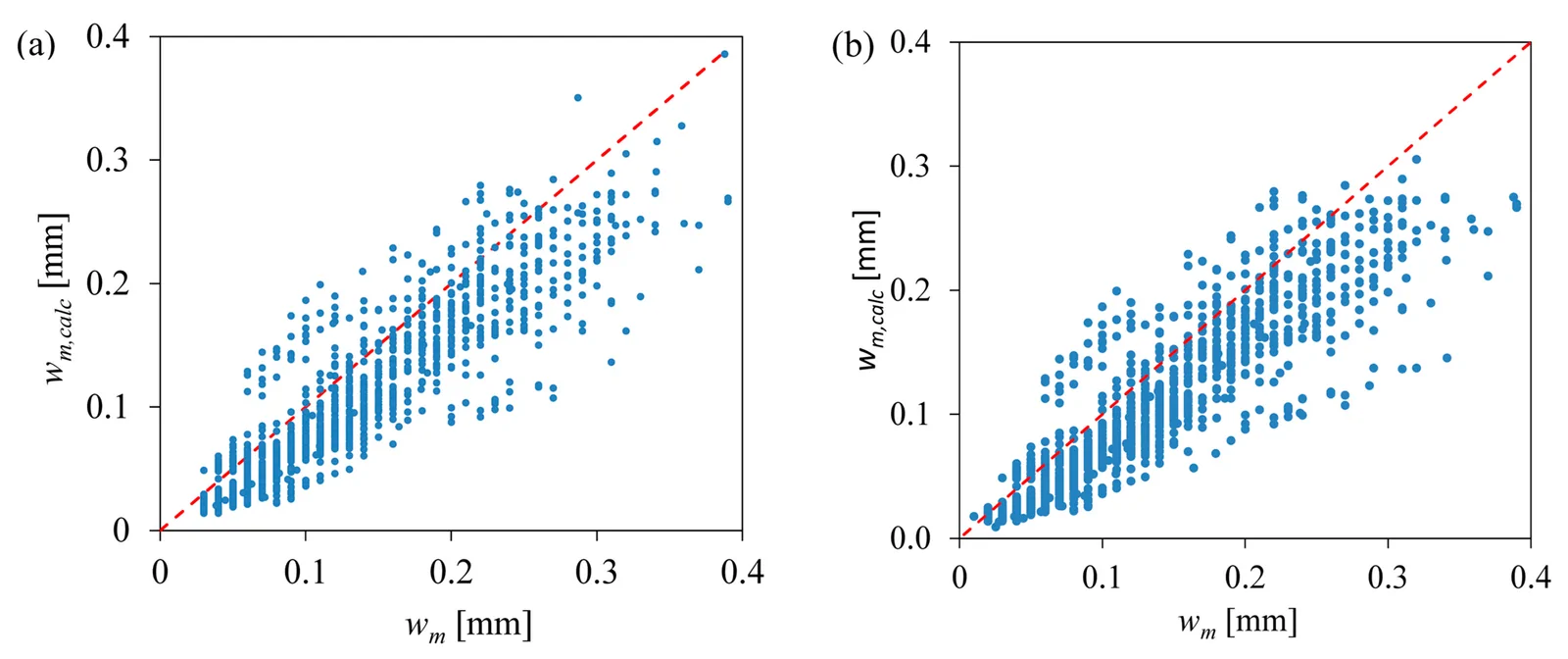
Comparison between measured and calculated mean crack widths: (**a**) EC2023; (**b**) MC2020.

Both formulations capture the overall trend of the experimental data; however, most predictions fall below the line of agreement (1:1), indicating a systematic underestimation of mean crack widths. For EC2023, the error metrics are MAE = 0.033 mm, RMSE = 0.041 mm, MAPE = 31%, and (*R*^2^ = 0.72), with 89% of cases underestimated. MC2020 yields similar results: MAE = 0.033 mm, RMSE = 0.042 mm, MAPE = 31.4%, and (*R*^2^ = 0.70), with 89.5% of cases underestimated. The similar performance indicates that the observed underestimation is not specific only to the EC2023 formulation and supports examining how the adopted bond-transfer and cracking-stage assumptions represent the response of end-restrained members subjected to imposed strain.

### 4.2. Prediction of Upper-Tail Crack Width

In the second stage, the design crack widths calculated according to EC2023 [13] and MC2020 [43] were compared with the adopted 95th-percentile reference values (Figure 4a,b, respectively). For most observations, the reference was *w*_0.95,_*_approx_* obtained from Equations (1)–(3). Across the complete dataset, application of the global factors resulted in underestimation rates of approximately 47% for EC2023 (factor of 1.7) and 26.2% for MC2020 (factor of 2.0). Thus, MC2020 produced fewer underestimated cases but showed a more pronounced tendency towards overestimation. For the subset with empirical percentiles, these proportions increased to 60% and 73%, respectively. This difference confirms that the moderately lower-biassed approximation of *w*_0.95_ affects the apparent upper-tail performance. For both models, underestimation was more frequent for smaller crack widths. These results indicate that global multipliers do not fully resolve the discrepancies across the analysed crack-width range. While MC2020 is included as an additional benchmark, the subsequent analyses focus on the EC2023 model.

### 4.3. Error Distribution and Stress-Dependent Underestimation

The error distribution *e* = *w_max_*_,_*_cal_*_−_ *w*_0.95_ (Figure 5) is centred close to zero, with the highest frequency of cases occurring just below the line of equality, around −0.01 to −0.02 mm, and 47% of cases lying to the left of the zero-error line. This histogram indicates that the model frequently produces values below the estimated *w*_0.95_, which is particularly relevant because this quantity represents an estimated upper-tail reference rather than the directly measured maximum crack width. Most errors are concentrated near zero, showing that for a large proportion of specimens the model yields values close to the experimental results. However, the distribution is slightly skewed towards the negative side, with a visible cluster of small underestimations. Conversely, the positive tail extends further, suggesting that overestimation cases, though less frequent in the peak region, are more dispersed and occur predominantly for larger crack widths.

The database-level correlation analysis and subsequent ML interpretation indicate that reinforcement stress *σ_s_* is one of the key variables governing crack-width development. For this reason, *σ_s_* was adopted as the explanatory variable in the cumulative underestimation plot of the EC2023-based model (Figure 6). The results show a monotonic decrease in underestimation with increasing *σ_s_*: from 46.5% at 50 MPa, to less than 3% around *σ_s_* ≈ 330 MPa, and effectively 0% for *σ_s_* ≥ 412 MPa. This confirms that the tendency of the original EC2023 formulation to produce values below the adopted *w*_0.95_ reference is concentrated in the lower stress range, where the standard spacing assumptions are less representative of the observed response.

### 4.4. Internal Sensitivity Analysis of the EC2023 Formulation

An internal sensitivity analysis of the EC2023 crack-width formulation was carried out to identify which input parameters primarily control the calculated crack width within the adopted code model. This analysis was not intended as an external validation against experimental data, but as a parametric investigation of the structure of the formulation itself. Particular attention was paid to reinforcement stress because this parameter is not directly defined by the code for end-restrained members subjected to imposed strain.

A synthetic dataset of 5000 admissible cases was generated using the NumPy random-number generator default_rng with a fixed seed of 42. Uniform sampling was adopted to explore the admissible parameter domain of the EC2023 formulation rather than to reproduce the frequency distribution of the experimental database. Concrete class was sampled with equal probability from C16/20 to C80/95, and bar diameter was sampled with equal probability from ϕ ∈ {12, 14, 16, 18, 20, 22, 25} mm. For each sampled combination, *A_s_* = π*ϕ*^2^/4 and the admissible range of the side length *h* was determined from the joint geometrical and reinforcement constraints. For the centrally reinforced square sections *c* = (*h*−*ϕ*)/2, while the reinforcement ratio was required to satisfy:

(6) $$\frac{f_{ct,eff}}{f_{yk}}\;\leq \;\rho \;=\;\frac{A_{s}}{h^{2}}\;\leq \;0.04$$

where *f_yk_* = 500 MPa. Consequently,

(7) $$h_{min}=max(100,\;\phi +30,\;\sqrt{\frac{A_{s}}{0.04}})$$

and

(8) $$h_{max}=min(\phi +100,\;\sqrt{\frac{A_{s}\;f_{yk}}{f_{ct,eff}}})$$

The side length *h* was sampled from a continuous uniform distribution over this case-specific admissible interval. Reinforcement stress was subsequently sampled from:

(9) $$\sigma_{s}\;∼\;U(\sigma_{s,min},\;500),\;\sigma_{s,min}\;=\;\frac{f_{ct,eff}\;h^{2}}{A_{s}}.$$

The quantities *A_s_*, *c*, *ρ*, *f_ct_*_,_*_eff_*, and *E_cm_* were therefore derived rather than independently sampled variables. The resulting dataset covered *h* = 100.0–125.0 mm.

For the resulting synthetic dataset, Pearson correlation coefficients were calculated between the input parameters and the crack width *w_k_*_,_*_cal_* predicted by the EC2023 formulation. The results are summarised in Table 3.

The calculated crack width was governed primarily by reinforcement stress. The Pearson correlation coefficient between *σ_s_* and *w_k_*_,_*_cal_* was *r* = 0.799, which was markedly higher than for all other analysed variables. In contrast, the influence of concrete-related parameters was weak and negative. The correlation coefficients obtained for *f_ct_*_,_*_eff_* and *E_cm_* were both *r* = −0.065. The negative sign is consistent with the EC2023 strain-difference term, in which higher tensile strength reduces the calculated difference between mean reinforcement and concrete strains. However, these two parameters should not be interpreted independently because both were derived from the concrete class and are therefore strongly correlated.

The geometrical and reinforcement-related variables exhibited substantially weaker correlations with *w_k_*_,_*_cal_*: *r* = 0.180 for *h*, *r* = 0.159 for *A_s_*, *r* = 0.159 for *ϕ*, and *r* = 0.083 for *c*. These correlations are conditional on the adopted sampling ranges and admissibility constraints. The narrow range of section dimensions and the dependencies between inputs (particularly *A_s_* and *ϕ*, and *c* = (*h* − *ϕ*)/2) prevent the correlations from being interpreted as independent measures of parameter influence. Consequently, the low correlations obtained for geometrical variables do not establish that cover, member size, or reinforcement arrangement are generally of secondary importance.

This ranking should not be extrapolated to beams, slabs, walls, or other non-square sections. Moreover, the proposed empirical model is restricted to members restrained at opposite ends within the investigated geometrical and loading domains.

Within the adopted sampling ranges and constraints, reinforcement stress exhibited the strongest correlation with the calculated crack width. This finding supports the use of *σ_s_* as a key intermediate variable when applying the EC2023 formulation to the investigated imposed-strain cases and motivates the semi-empirical *σ_s_*(*ε_imp_*) relationship introduced later in this study.

## 5. ML-Supported Diagnostic Analyses

### 5.1. Direct ML Prediction of Mean Crack Width from Imposed Strain

A separate machine-learning benchmark tested whether the mean crack width *w_m_* can be predicted directly from imposed-strain-level variables. In this benchmark, measured reinforcement stress *σ_s_* was deliberately excluded from the input vector. The purpose was to assess whether *ε_imp_*, combined with material, geometrical, and reinforcement-related parameters, contains sufficient information to reproduce the mean cracking response within the analysed experimental domain.

The input vector consisted of nine parameters: number of bars *n*, concrete cover *c*, bar diameter *ϕ*, mean tensile strength of concrete *f_ctm_*, mean modulus of elasticity of concrete *E_cm_*, concrete tensile area *A_ct_*, bond coefficient *k_b_*, reinforcement ratio *ρ*, and imposed strain *ε_imp_*. The target variable was the experimentally measured mean crack width *w_m_*. Thus, the model used only deformation-, material-, geometrical-, reinforcement-, and bond-related quantities available without measured *σ_s_*.

Figure 7 compares the measured and predicted mean crack widths for the four regression models: Linear Regression, Random Forest, Gradient Boosting, and CatBoost. The linear model captures the general trend of the data, but its larger scatter confirms that the relationship between imposed strain, reinforcement layout, material properties, and crack width cannot be fully represented by a purely linear approximation. The tree-based models show a visibly tighter concentration of points around the line of equality, indicating their ability to capture nonlinear interactions between the governing variables. Among them, Gradient Boosting achieved the best testing performance, with *R*^2^ = 0.914, RMSE = 0.026 mm, and MAE = 0.019 mm (Table 4). CatBoost also performed well, with *R*^2^ = 0.906, RMSE = 0.027 mm, and MAE = 0.018 mm, whereas Random Forest showed slightly lower testing accuracy despite its very high training accuracy, suggesting a greater tendency towards overfitting.

The training and testing metrics show that excluding measured *σ_s_* does not prevent accurate prediction of the mean crack width. This result is important from an engineering point of view because it demonstrates that the available imposed-strain-level variables can reproduce the mean cracking response with high accuracy within the analysed experimental domain.

### 5.2. SHAP-Based Interpretation of the ε_Imp_-Based Crack-Width Model

Figure 8 presents the normalised feature importance based on mean absolute SHAP values for the ML models predicting the mean crack width *w_m_*. For the tree-based models, *ε_imp_* is clearly identified as the dominant predictor, with the highest normalised mean |SHAP| value among the input variables. This confirms that imposed strain provides the primary deformation-related information needed to predict *w_m_* when measured *σ_s_* is not included in the model.

The remaining variables, including *ρ_eff_*, *ϕ*, c, *f_ctm_*, *E_cm_*, *A_ct_*, and *k_b_*, have lower but still non-negligible importance. Their contribution can be interpreted as a secondary modification of the imposed-strain response, reflecting the effects of reinforcement ratio, bar diameter, concrete tensile resistance, bond transfer, and crack spacing. The different importance pattern obtained with Linear Regression reflects its limited ability to capture these nonlinear interactions.

Figure 9 provides a more detailed SHAP-based interpretation of the best-performing Gradient Boosting model. The SHAP values for *ε_imp_* show a clear and physically consistent ordering: low imposed-strain values are concentrated mainly on the negative side of the SHAP scale, whereas higher values progressively shift towards positive SHAP contributions. Thus, increasing *ε_imp_* systematically increases the predicted *w_m_*, which is consistent with the cracking mechanism of end-restrained members subjected to imposed strain.

The present ML benchmark should therefore be interpreted as a diagnostic reference rather than as a replacement for a mechanics-based crack-width formulation. Its role is to quantify the prediction accuracy attainable from imposed-strain-level variables and to provide a reference for assessing the original and modified EC2023-based models.

### 5.3. Machine-Learning Interpretation of the ε_Imp_–σ_s_ Relationship

The relationship between imposed strain and reinforcement stress was analysed using ML models to support the subsequent formulation of a semi-empirical *σ_s_*(*ε_imp_*) model. The aim was to verify whether *σ_s_* can be estimated from imposed strain and selected material, geometrical, and reinforcement-related parameters, and to identify the variables needed for a physically meaningful relationship for estimating reinforcement stress.

The predictive performance of the analysed models is summarised in Table 5. All models reproduced the general *ε_imp_*-*σ_s_* relationship with high accuracy, but the nonlinear tree-based algorithms clearly outperformed Linear Regression. Gradient Boosting provided the best overall testing performance, with *R*^2^ = 0.987, RMSE = 13.9 MPa, and MAE = 10.2 MPa. Random Forest and CatBoost gave similarly high accuracy, but Gradient Boosting offered the most favourable balance between accuracy, generalisation, and interpretability. It was therefore selected as the reference model for the detailed interpretation of the *ε_imp_*-*σ_s_* relationship. Here, Gradient Boosting was selected only as the reference model for interpreting the *ε_imp_*-*σ_s_* relationship and for supporting the subsequent semi-empirical stress model.

Figure 10 compares the measured reinforcement stresses with the values predicted by the Gradient Boosting model. The data points are closely concentrated around the line of agreement for both training and testing data, confirming the high predictive capability of the model. The testing results show that the nonlinear *ε_imp_*-*σ_s_* relationship can be captured with high accuracy within the analysed experimental domain, with somewhat larger deviations at higher stress levels.

The SHAP interpretation of the Gradient Boosting model is presented in Figure 11. The ranking of variables is consistent with the physical structure expected for the stress-estimation problem: *ε_imp_* and *ρ_eff_* provide the main deformation-related contribution, while *ϕ*, and *f_ctm_* represent the most relevant secondary variables. Their influence is mechanically meaningful because *ρ_eff_* affects the stiffness of the reinforced section and post-cracking force redistribution, *ϕ* is related to bond-transfer conditions and reinforcement surface-to-area ratio, and *f_ctm_* controls the cracking-force level. The partly discrete SHAP patterns observed for *ρ_eff_* and *ϕ* reflect the limited number of reinforcement layouts and bar diameters in the source experimental programmes. The remaining variables, including *c*, *E_cm_*, *A_ct_*, *k_b_*, and *n*, have smaller contributions within the analysed experimental domain.

Overall, the ML analysis identified *ε_imp_* as the dominant predictor of reinforcement stress and *ρ_eff_*, *ϕ* and *f_ctm_* as the most relevant secondary variables within the analysed domain. This ranking was used to screen the candidate inputs to the semi-empirical model presented in Section 7.

## 6. Bond- and Stage-Sensitive Modification of the EC2023 Crack-Width Model

### 6.1. Reinterpretation of the EC2023 Spacing Term

The stress-dependent underestimation identified in Section 4.3 indicates that the constant bond-related term embedded in the EC2023 spacing expression does not provide uniform accuracy over the full experimental response range. In particular, the lower stress range requires a larger effective spacing contribution than that implied by the nominal EC2023 formulation. This motivates a stress-dependent reinterpretation of the effective bond-transfer contribution implicitly included in the crack-spacing term. In EC2023, unlike in EC2:2004, the coefficients commonly denoted *k*_1_ and *k*_4_ are not written explicitly, but they remain embedded in the factor 1/7.2 used in the spacing expression. If *k*_4_ = 0.25 is adopted, the corresponding value is *k*_1_ = 0.56. This is lower than the value of 0.80 adopted for ribbed bars in EC2:2004 and follows from the simplifying assumption of a constant ratio *τ_b_*_,_*_m_*/*f_ctm_* = 1.8, consistent with the fib Model Code 2010 formulation [44]. Detailed analyses of the new Eurocode crack-width provisions and related models are available in recent studies [21,22,23,24].

The variability of this effective coefficient can be interpreted with reference to bond-slip theory. Based on the bond-transfer concepts discussed by König and Tue [45] and Marti et al. [46], the mean bond stress associated with very small crack widths is substantially lower than that corresponding to more developed cracks. For example, using the relationships in [45], the ratio of mean bond stresses for *w_k_* = 0.3 mm to *w_k_* = 0.03 mm is approximately 2.66 for C30/37 concrete and *σ_sr_*/*σ_s_*_2_ = 0.3. In contrast, the ratio between *w_k_* = 0.5 mm and *w_k_* = 0.3 mm is approximately 0.86. Because the spacing contribution is inversely related to the effective bond-transfer capacity, these values indicate that a constant bond-related coefficient cannot represent the full crack-width range with uniform accuracy. In this study, the nominal value *k*_1_ = 0.56 was therefore treated as representative of intermediate crack widths, approximately *w_k_* = 0.2–0.4 mm, while deviations from this level were introduced through a stress-dependent correction. This interpretation remains approximate because the effective coefficient may also depend on concrete strength, reinforcement stress, and bar diameter.

The stress-dependent variation in *k*_1_ was then identified by calibrating the EC2023 model against the measurement-level database. The calibration was performed under the requirement that, for each trial value of *k*_1_, all predictions satisfy *w_calc_* ≥ *w*_0.95_. Under this condition, the required variation in *k*_1_ was obtained, as shown in Figure 12 (blue curve); its shape and range are consistent with expectations from the König and Tue model [45]. The red line denotes the constant value *k*_1_ = 0.56 currently adopted in EC2023. For engineering use, a power-law approximation of this coefficient was adopted as follows:

(10) $$k_{1}=0.56\left(0.85+1.65{\left(\frac{500-\sigma_{s}/MPa}{450}\right)}^{1.805}\right)$$

Equation (10) was calibrated for reinforcement stresses ranging from approximately 50 to 500 MPa. Outside this range, the boundary values *k*_1_ = 1.40 for *σ_s_* < 50 MPa and *k*_1_ = 0.48 for *σ_s_* > 500 MPa may be adopted to avoid an extrapolation of the empirical relationship. However, crack-width predictions obtained outside the calibrated stress range have not been validated.

The physical meaning of this correction follows from the role of *k*_1_ in the simplified crack-spacing term. In an EC-type stress-transfer idealisation, this coefficient is approximately proportional to the inverse normalised effective mean bond stress, *k*_1_ ∝ *f_ctm_*/*τ_bm_*_,_*_eff_*; hence, a decrease in *k*_1_ represents a greater degree of bond activation. Interpreted in this way, Equation (10) corresponds to *τ_bm_*_,_*_eff_*/*f_ctm_* ≈ 1/*k*_1_, increasing from approximately 0.71 at *σ_s_* = 50 MPa to 1.79 at *σ_s_* = 381 MPa and 2.10 at *σ_s_* = 500 MPa. This direction is qualitatively consistent with established bond-transfer models for ribbed bars. Increasing reinforcement stress is accompanied by increasing crack opening and relative slip; over the ascending branch of a bond–slip relationship, larger slip activates higher local and average bond stresses. The associated stress-transfer zones, tension-stiffening contribution and crack formation evolve until a more stabilised cracking pattern is approached [46,47,48,49].

Equation (10), however, should not be interpreted as a local bond–slip law or as a direct measurement of bond stress. Reinforcement stress is used as a practical indicator of cracking development, whereas local bond is governed by slip and also depends on concrete properties, bar diameter and rib geometry, cover, casting conditions, crack spacing and possible splitting damage [44,50,51]. Because *k*_1_(*σ_s_*) was identified by inverse calibration against global crack-width observations, it represents an effective correction of the bond/spacing contribution and may also absorb effects of the evolving cracking pattern and residual model simplifications. Accordingly, the agreement with bond–slip theory is qualitative in direction; the exponent and numerical coefficients in Equation (10) are database-calibrated quantities and are applicable only within the stated calibration range.

It should be noted that Equation (10) was identified using the same measurement-level database that is subsequently used to assess the performance of the modified EC2023 formulation in Section 6.3 and Section 6.4. Therefore, the results presented in these sections constitute an internal calibration assessment rather than independent validation. They quantify the agreement attainable within the experimental domain used for parameter identification and should not be interpreted as evidence of predictive performance for independent experimental datasets.

### 6.2. Stage-Dependent Correction of the Strain-Difference Term

In EC2023 [13], *k_t_* is an integration factor accounting for the variation in reinforcement and concrete strains over two transfer lengths. Within the mean strain-difference expression, it controls the contribution of tensile concrete between cracks and therefore represents, in a simplified manner, the tension-stiffening effect. A higher value of *k_t_* produces a greater reduction in the mean reinforcement–concrete strain difference, whereas a lower value represents a smaller contribution of tensile concrete between cracks. Although EC2023 assigns *k_t_* according to load duration, the effective tension-stiffening contribution may also vary with the stage of crack development, which can be represented by a measure of strain or reinforcement stress relative to its value at crack initiation. Accordingly, an effective stage-dependent coefficient was introduced in the form:

(11) $$k_{t,eq}=0.43+0.15\frac{\sigma_{cr}}{\sigma_{s}}$$

The cracking stress *σ_cr_* was calculated once for each specimen as an equivalent reinforcement stress corresponding to the nominal tensile force required to initiate cracking:

(12) $$\sigma_{cr}=\frac{F_{cr}}{A_{s}}=\frac{f_{ctm}\cdot A_{c}}{A_{s}}$$

where *f_ctm_* is the experimentally determined mean tensile strength of concrete for the relevant test sample, *A_c_* is the gross concrete cross-sectional area, and *A_s_* is the total area of longitudinal reinforcement. Accordingly, *σ_cr_* is constant for a given specimen and is not recalculated at successive loading stages. Therefore, *σ_cr_* should be interpreted as a consistent specimen-level reference quantity rather than as a directly measured local reinforcement stress at crack initiation. The sensitivity of the coefficient to this definition can be expressed as:

(13) $${∆k}_{t,eq}=0.15\frac{{∆\sigma }_{cr}}{\sigma_{s}}=\left(k_{t,eq}-0.43\right)\frac{{∆\sigma }_{cr}}{\sigma_{s}}$$

Thus, uncertainty in *σ_cr_* affects only the variable component *k_t_*_,_*_eq_* − 0.43. For the range *k_t_*_,_*_eq_* = 0.48–0.80 obtained in the analysed database, a ±10% change in *σ_cr_* produces an absolute change in *k_t_*_,_*_eq_* of approximately ±0.005 to ±0.037, corresponding to approximately 1.0–4.6% of its value. The sensitivity is highest near crack initiation and decreases with increasing reinforcement stress.

The stage dependence introduced in Equation (11) is consistent with the mechanics of progressive cracking described by Schlicke et al. [20]. During the crack-formation stage, bond transfers tensile force from the reinforcement to the concrete between cracks. Consequently, the tensile stress carried by the concrete reduces the mean reinforcement strain relative to that of a bare bar. As the imposed strain increases, additional cracks form, reinforcement–concrete slip develops, bond stresses are redistributed, and the crack pattern progressively approaches stabilisation. Once no further cracks can form within the available transfer lengths, subsequent deformation is accommodated predominantly by widening of the existing cracks. Thus, although tensile stresses may continue to be transferred to the concrete between cracks, their relative contribution to the mean reinforcement–concrete strain difference progressively decreases as the reinforcement stress increases.

The ratio *σ_cr_*/*σ_s_* was adopted as a dimensionless proxy for this evolution. It is comparatively high near crack initiation and decreases as the reinforcement stress increases beyond the cracking-stage value. Equation (11) therefore assigns a higher *k_t_*_,_*_eq_* during the early cracking stages and approaches the limiting value of 0.43 at more advanced stages. This limiting value is numerically close to the value *k_t_* = 0.4 specified in EC2023.

The linear form was selected as the simplest monotonic relationship capable of representing the systematic variation in the residuals with the cracking-stage indicator after modification of the crack-spacing term. The coefficients 0.43 and 0.15 were determined by least-squares calibration against the experimental database. Accordingly, Equation (11) should be regarded as an effective, phenomenological correction. Its physical interpretation is supported indirectly by the observed evolution of the cracking response, whereas direct validation against local bond and slip measurements remains a subject for further research.

### 6.3. Internal Calibration Performance for Mean Crack Width

Figure 13 compares the experimentally measured mean crack widths *w_m_* with the mean values predicted by the modified EC2023 model. Compared with the unmodified formulation, the scatter shows a visibly tighter concentration of points around the line of agreement. The goodness-of-fit metrics improve to MAE = 0.022 mm, RMSE = 0.032 mm, and MAPE = 21%, while the coefficient of determination increases to *R*^2^ = 0.83. The proportion of underestimated cases decreases from 89% in the original model to 41.5%, indicating a substantial reduction in the systematic bias. Some scatter remains, particularly for larger crack widths. Within the calibration dataset, however, the modified formulation provides markedly better agreement with the experimentally measured mean crack widths than the original EC2023 model.

### 6.4. Internal Calibration Performance for Upper-Tail and Maximum Crack Width

The internal calibration performance of the modified EC2023 model was then assessed against the adopted 95th-percentile crack-width reference (Figure 14). Across the complete dataset, the proportion of underestimated cases decreased from approximately 47% for the original formulation to 3.4% after correction. However, this result is influenced by the predominance of *w*_0.95,_*_approx_*, which was shown in Section 3.4 to be moderately lower-biassed. For the subset with *w*_0.95,_*_emp_* determined directly from complete crack-width distributions, the underestimation rate decreased from 60% to 30%. Thus, the modification substantially improves the upper-tail agreement, although the results based on *w*_0.95,_*_approx_* should not be interpreted as evidence of statistical conservatism.

A similar trend was obtained when the calculated maximum crack widths were compared directly with the measured *w_max_* values (Figure 15). In the direct comparison with the measured *w_max_*, only 5.5% of the observed maxima were underestimated. For the underestimated cases, the mean relative underestimation was 11.2%, corresponding to a mean absolute deficit of 0.023 mm. Although the scatter was naturally larger than for the mean crack widths, the comparison confirms that the proposed modification also provides a satisfactory prediction of the upper bound of crack width.

Although the modified EC2023 formulation substantially improves the prediction of mean and upper-tail crack widths, it still requires *σ_s_* as an input. To obtain a complete imposed-strain-based procedure, the modified crack-width model must therefore be supplemented with a relationship for estimating *σ_s_* from *ε_imp_* and selected material-reinforcement parameters.

## 7. Semi-Empirical Stress Model and Complete Modified EC2023 Procedure

### 7.1. Formulation of the Semi-Empirical σ_S_(ε_imp_) Model

The semi-empirical model was developed in three stages. First, the ML and SHAP analyses presented in Section 5.3 were used to identify the variables carrying the greatest predictive information for reinforcement stress. This screening indicated *ε_imp_* and *ρ_eff_* as the dominant variables, followed by *ϕ*, and *f_ctm_*. Second, these variables were incorporated into a compact relationship partly guided by the expected behaviour of restrained cracking. Third, the detailed form of the correction terms and their coefficients were determined by regression against the analysed experimental database.

The adopted form of the equation has a partial mechanical basis. The first term of the model refers to the steel stress resulting from force equilibrium in the cross-section at the moment when the tensile strength of concrete is reached. The correction coefficient (0.806) applied to this term accounts for force redistribution and partial unloading of the member after cracking. The second term of the equation describes the linear increase in reinforcement stress caused by increasing imposed strain, whereas the third term accounts for the nonlinear component of this increase. The dependence on *ρ_eff_* and *ϕ*/*ρ_eff_* additionally enables the influence of reinforcement intensity and stress-transfer conditions between concrete and steel to be considered. Finally, the reinforcement stress is calculated as:

(14) $$\frac{\sigma_{s,calc}}{MPa}=0.806\frac{100f_{ctm}/MPa}{\rho_{eff}/\%}+\frac{\varepsilon_{imp}}{‰}\left(42.135+11.162\frac{\rho_{eff}}{\%}+6.043\frac{\phi /mm}{\rho_{eff}/\%}\right)+7.104{\left(\frac{\varepsilon_{imp}}{‰}\right)}^{2}$$

### 7.2. Calibration-Fit Assessment of the Semi-Empirical Reinforcement-Stress Model

All quantities should be substituted using the units applied during model identification: *σ_s_*_,_*_calc_* in MPa, *ϕ* in mm, *f_ctm_* in MPa, *ρ_eff_* in %, and *ε_imp_* in ‰. The comparison between calculated and measured values for the database used in coefficient identification is presented in Figure 16. All retained observations were pooled and used jointly to determine a single set of regression coefficients; no grouped hold-out or source-wise calibration–validation split was applied. The data points are concentrated around the line of agreement *σ_s_*_,_*_calc_* = *σ_s_*_,_*_meas_*. For this calibration dataset, the model achieved *R*^2^ ≈ 0.945, RMSE ≈ 26.6 MPa, and MAE ≈ 18.2 MPa. These indicators therefore quantify the agreement between calculated and measured values within the database used for model identification and should not be interpreted as measures of independent predictive performance.

The proposed relationship is semi-empirical and was identified for the analysed dataset of reinforced concrete members subjected to imposed strain. Therefore, its application should be limited to the parameter range used in model calibration, i.e., bar diameter *ϕ* = 10–25 mm, mean tensile strength of concrete *f_ctm_* = 2.13–4.69 MPa, effective reinforcement ratio *ρ_eff_* = 0.785–3.6%, and imposed strain *ε_imp_* = 0.061–1.9‰. In the analysed dataset, this corresponded to a reinforcement stress range *σ_s_* of approximately 49–493 MPa. Extrapolation beyond this range may lead to increased uncertainty. In particular, the model should not be treated as a universal code-based relationship, but rather as a regression-based expression with partial mechanical justification intended to describe the analysed response domain.

The residual distribution of the proposed semi-empirical reinforcement-stress model was additionally examined with respect to imposed strain, as shown in Figure 17. The residuals are generally concentrated around the zero-error line, indicating that the model does not exhibit a pronounced global bias in the analysed strain range. No clear monotonic trend of *σ_s_* with increasing *ε_imp_* can be observed, suggesting that imposed strain is adequately represented in the adopted form. A somewhat larger scatter is visible at higher imposed strain levels, which may be attributed to the increasing variability of cracking stage, reinforcement stress redistribution, and bond conditions as the imposed deformation progresses. Occasional larger residuals are present, but they do not dominate the overall response. Therefore, the residual analysis supports the applicability of the proposed *σ_s_*(*ε_imp_*) relationship within the calibrated parameter range, while also confirming its semi-empirical character and the need to avoid extrapolation beyond the experimental domain used for model identification.

### 7.3. CatBoost-Based Verification Using Measured and Predicted Reinforcement Stress

An additional CatBoost-based analysis was carried out to examine how prediction of *w_m_* changes when reinforcement stress is used explicitly as the deformation-related input variable. This step complements the previous *ε_imp_*-based benchmark and verifies whether the stress-estimation stage preserves the information needed for crack-width prediction.

Only CatBoost is presented here, as it delivered the highest accuracy among all the evaluated models. The input vector was analogous to that used in the *ε_imp_*-based analysis, but imposed strain was replaced by reinforcement stress. Two variants were analysed. In the first variant, the experimentally measured reinforcement stress *σ_s_* was used as an input variable. In the second variant, *σ_s_* was replaced by the value predicted in the previous machine-learning analysis of the *ε_imp_*-*σ_s_* relationship. This enabled the influence of the stress-estimation step on the final crack-width prediction to be assessed.

Figure 18 compares the measured and CatBoost-predicted mean crack widths for the two analysed variants. In both cases, the data points are concentrated close to the line of equality, confirming that reinforcement stress contains substantial information relevant to crack-width prediction. However, the two variants show some differences. When measured *σ_s_* is used, testing performance reaches *R*^2^ = 0.851, with RMSE = 0.033 mm and MAE = 0.022 mm. When *σ_s_* predicted by the ML stress model is used instead, testing accuracy improves to *R*^2^ = 0.891, RMSE = 0.029 mm, and MAE = 0.02 mm. When comparing the prediction of mean crack width using measured versus ML-predicted reinforcement stress, the ML-predicted variant achieved slightly higher testing accuracy than the measured stress variant. This result should not be interpreted as evidence that predicted stress values are physically more accurate than direct sensor measurements, but rather reflects two underlying statistical and mechanical effects. Direct stress measurements in experimental ties exhibit local scatter caused by strain-gauge proximity to crack planes, micro-slip, and local concrete inhomogeneities. The intermediate ML stress model functions as a global physical filter, smoothing out high-frequency experimental noise and capturing the fundamental load-deformation trend. Because the ML-predicted stress is derived from the primary deformation demand and key geometric-material parameters, it preserves the complete mechanical information space of the direct deformation-based model. Because both the stress-estimation and crack-width models were evaluated using identical group-based specimen splits, this performance gain represents out-of-sample noise reduction rather than data leakage across datasets.

The comparison of the two CatBoost variants is summarised in Table 6. The model using measured reinforcement stress gives accurate predictions, but its testing performance is lower than that obtained in the earlier direct *ε_imp_*-based CatBoost model, for which *R*^2^ = 0.906, RMSE = 0.026 mm, and MAE = 0.019 mm were obtained. This confirms that imposed strain, when combined with material, geometrical, and reinforcement-related variables, is sufficient to reproduce the mean crack-width response with very high accuracy. At the same time, the variant based on ML-predicted *σ_s_* gives results close to the direct *ε_imp_*-based model. This is an important observation because it shows that the intermediate prediction of reinforcement stress does not lead to a significant loss of information in the subsequent crack-width prediction.

The SHAP-based interpretation of the CatBoost model using ML-predicted reinforcement stress provides a more detailed insight into the role of *σ_s_* in the final prediction of *w_m_* (Figure 19). In this variant, predicted reinforcement stress is identified as the dominant input variable. This trend is physically consistent with the cracking mechanism of end-restrained members: increasing reinforcement stress intensifies bond-related slip between reinforcement and concrete, leading to wider cracks.

The remaining variables, including concrete cover *c*, bar diameter *ϕ*, mean tensile strength *f_ctm_*, modulus of elasticity *E_cm_*, effective reinforcement ratio *ρ_eff_*, concrete tensile area *A_ct_*, bond coefficient *k_b_*, and number of bars *n*, have lower but still meaningful SHAP contributions. Their influence should be interpreted as a secondary modification of the stress-crack-width relationship, related mainly to crack spacing, bond transfer, reinforcement distribution, and material stiffness. The fact that the CatBoost model retains a clear, mechanically interpretable response when measured *σ_s_* is replaced by ML-predicted *σ_s_* is important because it indirectly confirms the usefulness of the preceding stress-prediction model as an intermediate component in the crack-width prediction chain.

Overall, the results show that ML-predicted reinforcement stress provides a mechanically consistent and practically applicable route for predicting the mean crack width. Although the direct *ε_imp_*-based CatBoost model remains slightly more accurate and avoids an intermediate stress-estimation step, the *σ_s_*-based variant is particularly relevant for the subsequent engineering interpretation. It demonstrates that once reinforcement stress is reliably estimated from imposed strain and material-geometrical parameters, it can be used successfully as the governing input variable for crack-width prediction. Therefore, this analysis supports the use of a *σ_s_*(*ε_imp_*) relationship in the modified EC2023-based calculation procedure.

### 7.4. Application of the σ_S_(ε_imp_) Model in the Modified EC2023 Procedure

In the final step, the semi-empirical reinforcement-stress model was incorporated into the modified EC2023 crack-width procedure. This produced a complete imposed-strain-based calculation route, in which *σ_s_* is first estimated from *ε_imp_* and selected material-reinforcement parameters and then introduced into the modified crack-spacing and strain-difference terms. The complete equation-by-equation implementation is provided in Section S1, while the main calculation sequence is summarised below.

First, reinforcement stress *σ_s_*_,_*_calc_* is estimated from imposed strain *ε_imp_* and selected material-geometrical parameters using Equation (14). The first term is related to the reinforcement stress corresponding to the cracking-force level, whereas the remaining terms describe the increase in reinforcement stress with imposed strain, including the influence of reinforcement intensity and bar diameter.

The second modification concerns the mean strain-difference term. The constant coefficient *k_t_* is replaced by an effective coefficient based on the ratio *σ_cr_*/*σ_s_*, which serves as a practical indicator of cracking development. In this way, the procedure accounts for stage-dependent tension stiffening while preserving the EC2023 calculation format.

Thus, the final calculation sequence consists of estimating *σ_s_*_,_*_calc_* from *ε_imp_*, *ϕ*, *f_ctm_*, and *ρ_eff_*, introducing this stress into the modified crack-spacing and strain-difference terms, and calculating either the mean crack-width estimate or the upper-tail/design crack-width value according to the modified EC2023 format. The proposed procedure therefore links imposed strain directly with crack-width prediction while preserving the general structure of the Eurocode crack-width formulation.

### 7.5. Comparison of the Code-Based Models, Modified EC2023 Procedures and ML Benchmarks

The final assessment compares the original EC2023 and MC2020 formulation, the modified EC2023 model using measured reinforcement stress, the complete modified procedure with *σ_s_* calculated from the proposed *σ_s_*(*ε_imp_*) relationship, and the ML benchmarks developed in the preceding sections. The purpose of this comparison is not only to evaluate prediction accuracy, but also to distinguish between regression performance, exceedance of the estimated upper-tail reference *w*_0.95_, performance relative to the measured maximum crack width *w_max_*, and practical applicability.

The original EC2023 and MC2020 formulations reproduced the general trend of the experimental data but showed a systematic underestimation bias, particularly in the lower reinforcement-stress range identified in Section 4.3. The bond- and stage-sensitive corrections substantially improved the response when measured *σ_s_* was used, especially for upper-tail crack-width prediction. Across the complete dataset, the underestimation rate relative to the adopted *w*_0.95_ reference decreased to 3.4%, while it decreased from 60% to 30% for the subset with *w*_0.95,_*_emp_*. The underestimation rate relative to *w_max_* was 5.5%.

The complete modified procedure further removes the need to prescribe *σ_s_* independently by estimating reinforcement stress from *ε_imp_*, *ϕ*, *f_ctm_*, and *ρ_eff_* before applying the modified EC2023 crack-width model (Figure 20, Table 7). This additional stress-estimation step did not substantially reduce the upper-tail performance of the modified model. Across the complete dataset, the underestimation rate relative to the adopted *w*_0.95_ reference was 1.76%. For the measured maximum crack width *w_max_*, the underestimation rate was 3.93%, with a mean deficit of 0.047 mm.

The ML benchmarks provide a useful reference for the attainable regression accuracy. The direct *ε_imp_*-based CatBoost model and the CatBoost model using ML-predicted *σ_s_* achieved higher accuracy in mean crack-width prediction than the engineering procedure.

However, their role in this study is diagnostic rather than prescriptive: they confirm the relevance of *ε_imp_* and support the intermediate *σ_s_*(*ε_imp_*) relationship, whereas the modified EC2023 procedure provides a transparent, code-compatible calculation format and substantially reduces the frequency of predictions below the adopted upper-tail reference. The main outcomes of the comparison are summarised in Table 7.

## 8. Conclusions

This study investigated the interface-controlled cracking response of end-restrained reinforced concrete members subjected to imposed strains and translated the observed bond- and stage-dependent behaviour into a mechanically interpretable, code-compatible crack-width prediction procedure. A measurement-level experimental database was compiled from available test results, comprising 137 reinforced concrete specimens and 1473 filtered observations corresponding to successive imposed-strain or loading stages. The following conclusions can be drawn:

1. The original EC2023 crack-width model reproduced the general trend of the experimental response, but showed a systematic tendency to underestimate crack widths because the embedded bond-transfer and crack-spacing assumptions were not sufficiently representative of the observed imposed-strain response. This confirmed that, within the analysed database, direct application of the standard formulation to end-restrained members subjected to imposed strain frequently produces crack-width values below the adopted experimental references.
2. The model deficiency was strongly stress-dependent. Underestimation was concentrated mainly in the low-to-intermediate reinforcement stress range and decreased progressively with increasing *σ_s_*, confirming that the accuracy of the EC2023 spacing assumptions varies with cracking development.
3. A bond- and stage-sensitive extension of the EC2023 formulation was therefore proposed. The modified spacing term introduces a stress-dependent bond contribution, while the mean strain-difference term is adjusted using *σ_cr_*/*σ_s_* as a practical indicator of cracking development. This preserves the general Eurocode format while improving its consistency over the calibrated response range.
4. Within the calibration database, the modified EC2023 model substantially improved agreement with the experimental data. When measured reinforcement stresses were used, the goodness of fit for mean crack width increased from *R*^2^ = 0.72 to *R*^2^ = 0.83. The underestimation rate relative to the adopted *w*_0.95_ reference decreased to 3.4% overall (and to 30% for the subset with *w*_0.95,_*_emp_*), while the corresponding rate for *w_max_* was 5.5%. This confirms that the proposed bond- and stage-sensitive correction substantially reduces the underestimation bias.
5. The ML analyses confirmed, in a diagnostic rather than prescriptive role, that imposed strain, reinforcement stress, and selected material–reinforcement parameters contain the key information needed to describe the cracking response. The direct *ε_imp_*-based CatBoost model provided a high-accuracy benchmark for mean crack-width prediction, while SHAP interpretation supported the mechanical relevance of *ε_imp_*, *ρ_eff_*, *ϕ* and *f_ctm_*.
6. The ML-supported analysis of the *ε_imp_*–*σ_s_* relationship provided the basis for a semi-empirical reinforcement-stress model. This model enables *σ_s_* to be estimated from imposed strain, bar diameter, concrete tensile strength and effective reinforcement ratio, thereby supplying the intermediate stress input required by the modified EC2023 crack-width procedure.
7. Incorporating the proposed *σ_s_*(*ε_imp_*) relationship into the modified EC2023 formulation resulted in a complete imposed-strain-based calculation procedure. Across the complete dataset, the calculated values equalled or exceeded the adopted *w*_0.95_ reference in 98.24% of the cases, although the underestimation rate was 30% for the subset with *w*_0.95,_*_emp_*. The corresponding underestimation rate for *w_max_* was 3.93%. Thus, the stress-estimation step did not compromise the upper-tail reliability of the modified crack-width model.

Overall, the results show that the crack-width response of end-restrained reinforced concrete under imposed strain can be represented more consistently when the Eurocode formulation is supplemented with bond- and stage-sensitive terms and with a semi-empirical reinforcement-stress model.

The proposed procedure remains code-compatible and mechanically interpretable, while substantially reducing the underestimation bias observed for the original formulation. Its application should, however, be limited to the calibration range of the present database: bar diameters of 10–25 mm, concrete tensile strengths of 2.13–4.69 MPa, effective reinforcement ratios of approximately 0.785–3.6%, imposed strains of 0.061–1.9‰, and reinforcement stresses of 49–493 MPa. Further validation on independent datasets, including broader material ranges, reinforcement layouts, member geometries, and long-term imposed deformations, is required before wider design application can be recommended.

A further potential extension concerns thermally-induced imposed strains. Numerical thermal analyses, such as that conducted by Bolina and Henn [52], demonstrate that the temperature fields within reinforced concrete members, including reinforcement temperatures, may vary substantially with concrete composition and thermal properties. Such thermal fields could provide input for a future thermomechanical extension of the present framework. This would require consideration of the spatially and temporally varying free thermal strains, restraint conditions, differential thermal expansion of concrete and reinforcement, and the temperature dependence of their mechanical and bond properties.

## Figures and Tables

**Figure 1:**
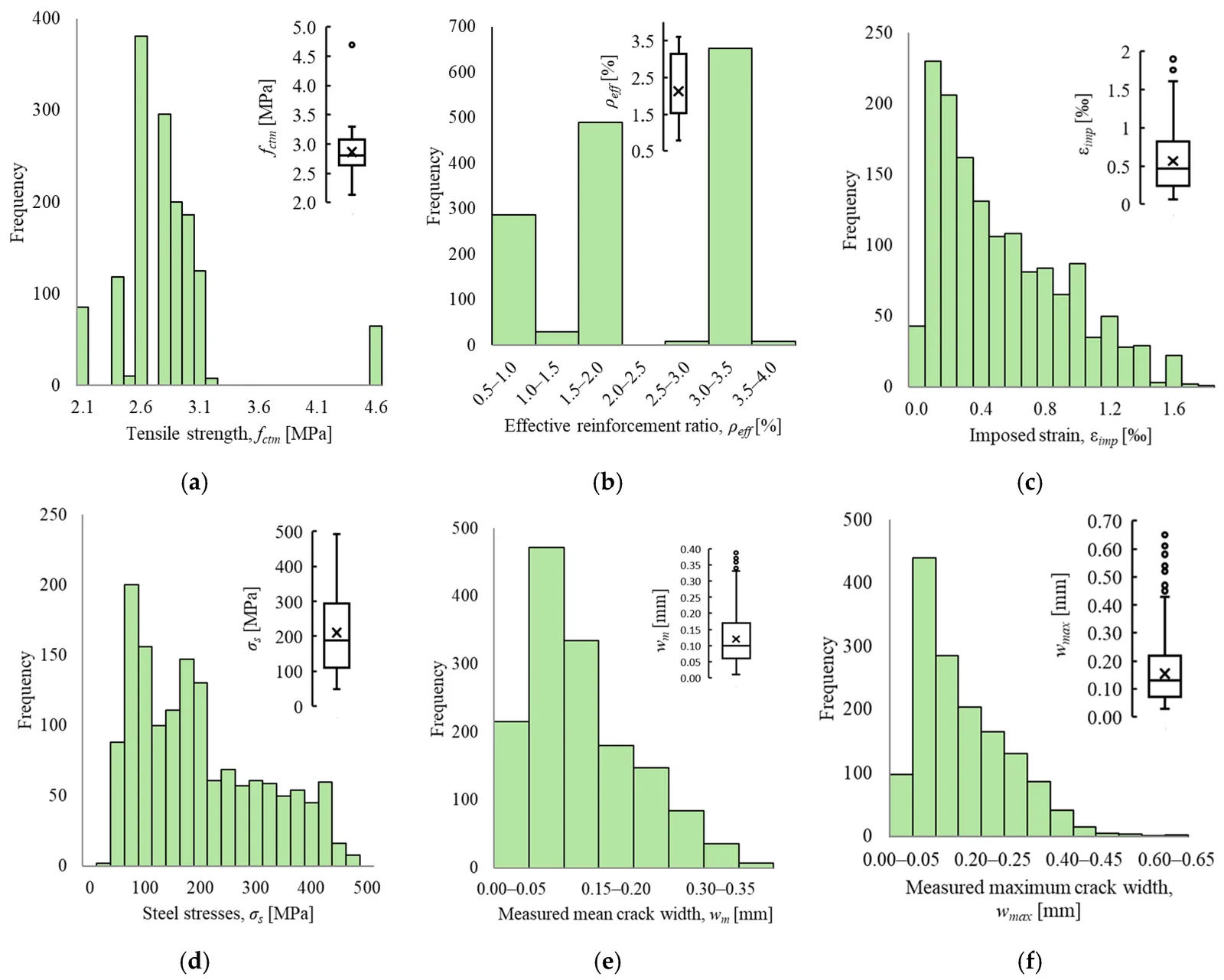
Distribution of selected variables in the experimental measurement database: (**a**) Mean tensile strength of concrete *f_ctm_*, (**b**) effective reinforcement ratio *ρ_eff_*, (**c**) imposed strain *ε_imp_*, (**d**) reinforcement stress *σ_s_*, (**e**) measured mean crack width *w_m_*, and (**f**) measured maximum crack width *w_max_*.

**Figure 2:**
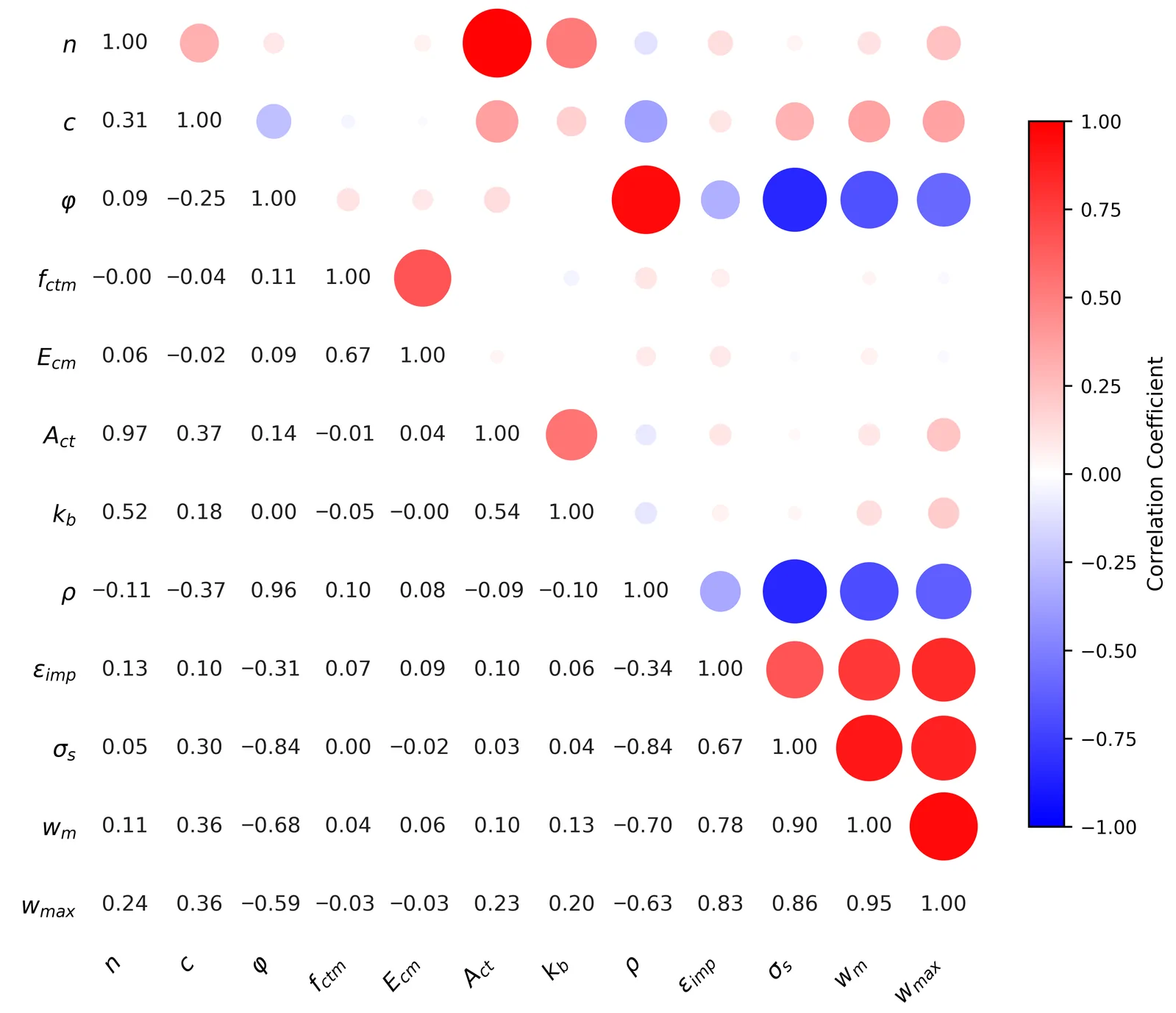
Pearson correlation matrix for the main variables included in the measurement-level database.

**Figure 4:**
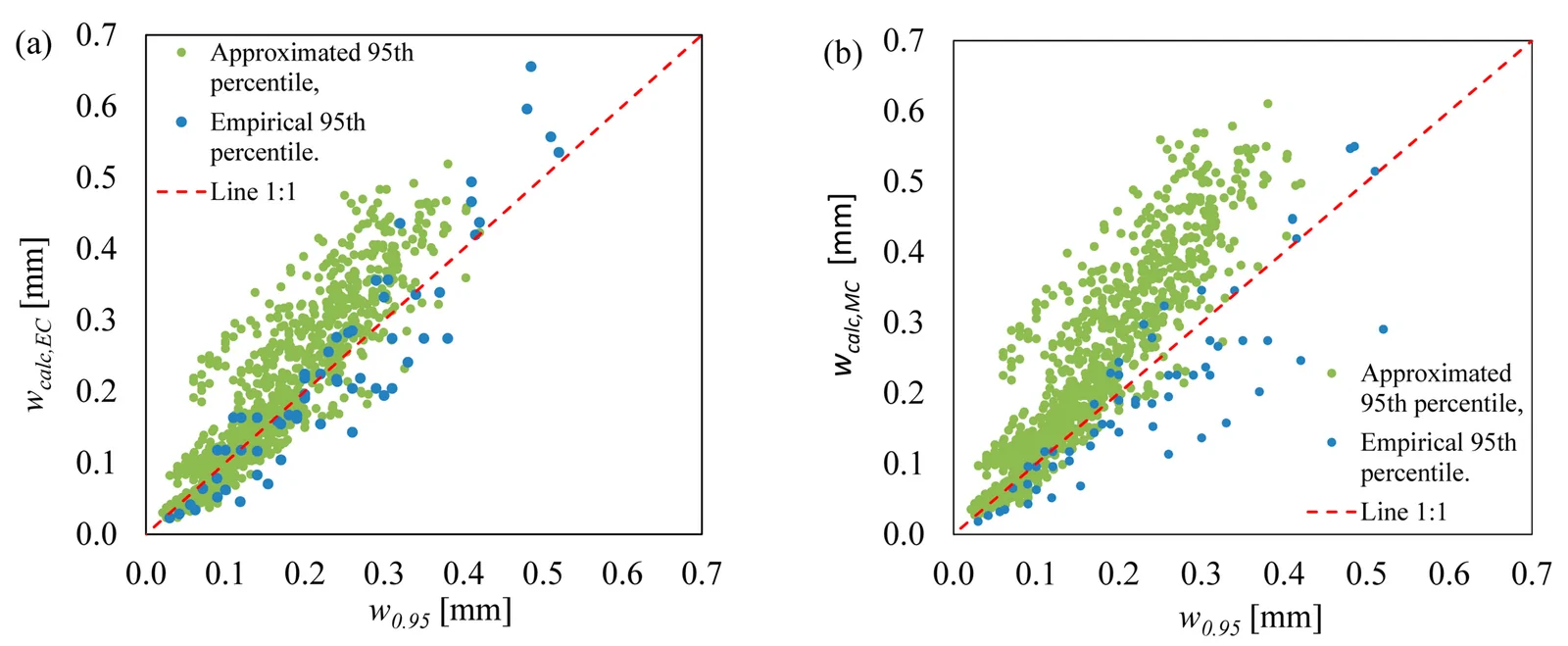
Comparison between the adopted 95th-percentile crack-width reference and calculated design crack widths: (**a**) EC2023; (**b**) MC2020. The approximated and empirical percentile values are distinguished by different symbols.

**Figure 5:**
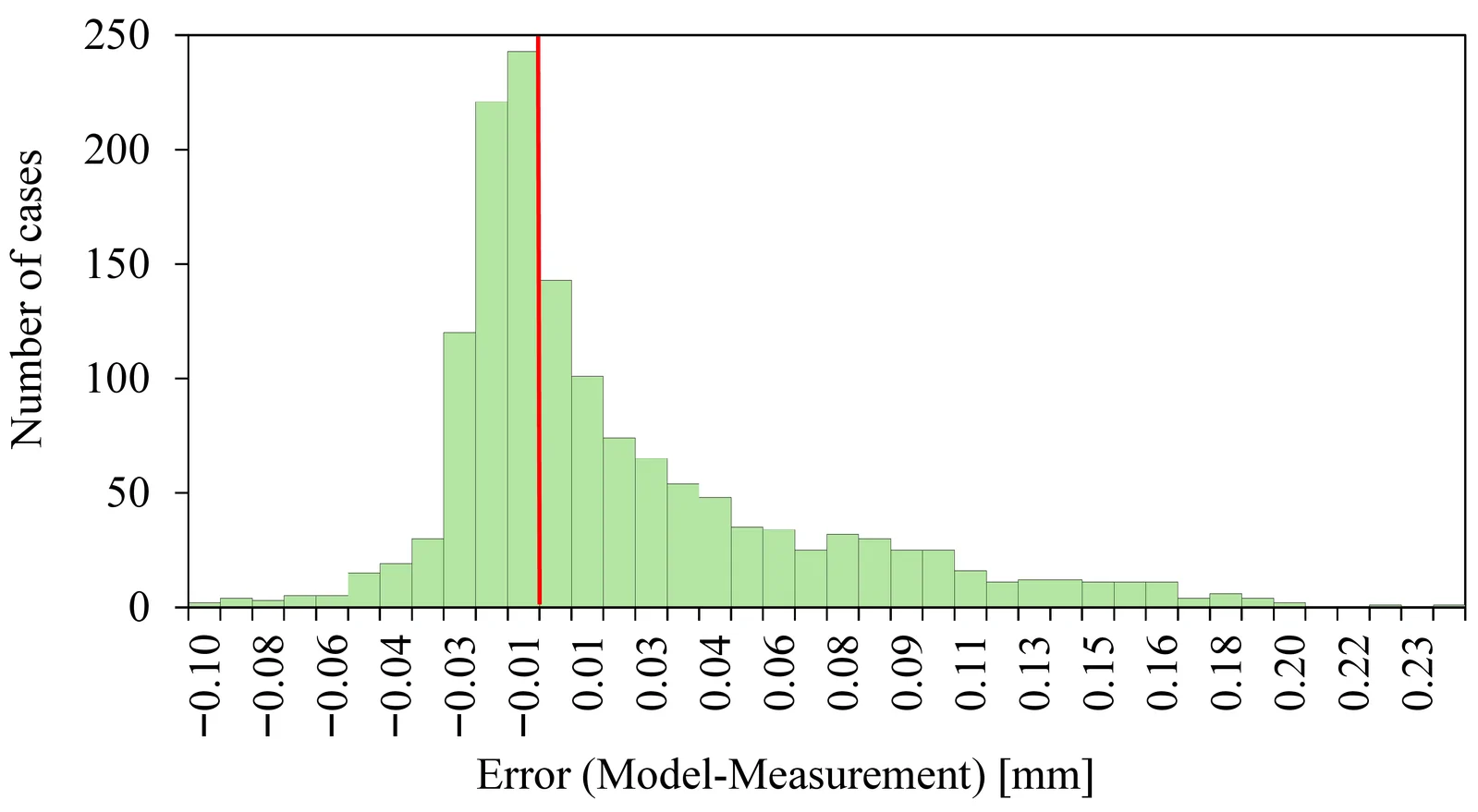
Histogram of signed prediction errors of the EC2023-based model.

**Figure 6:**
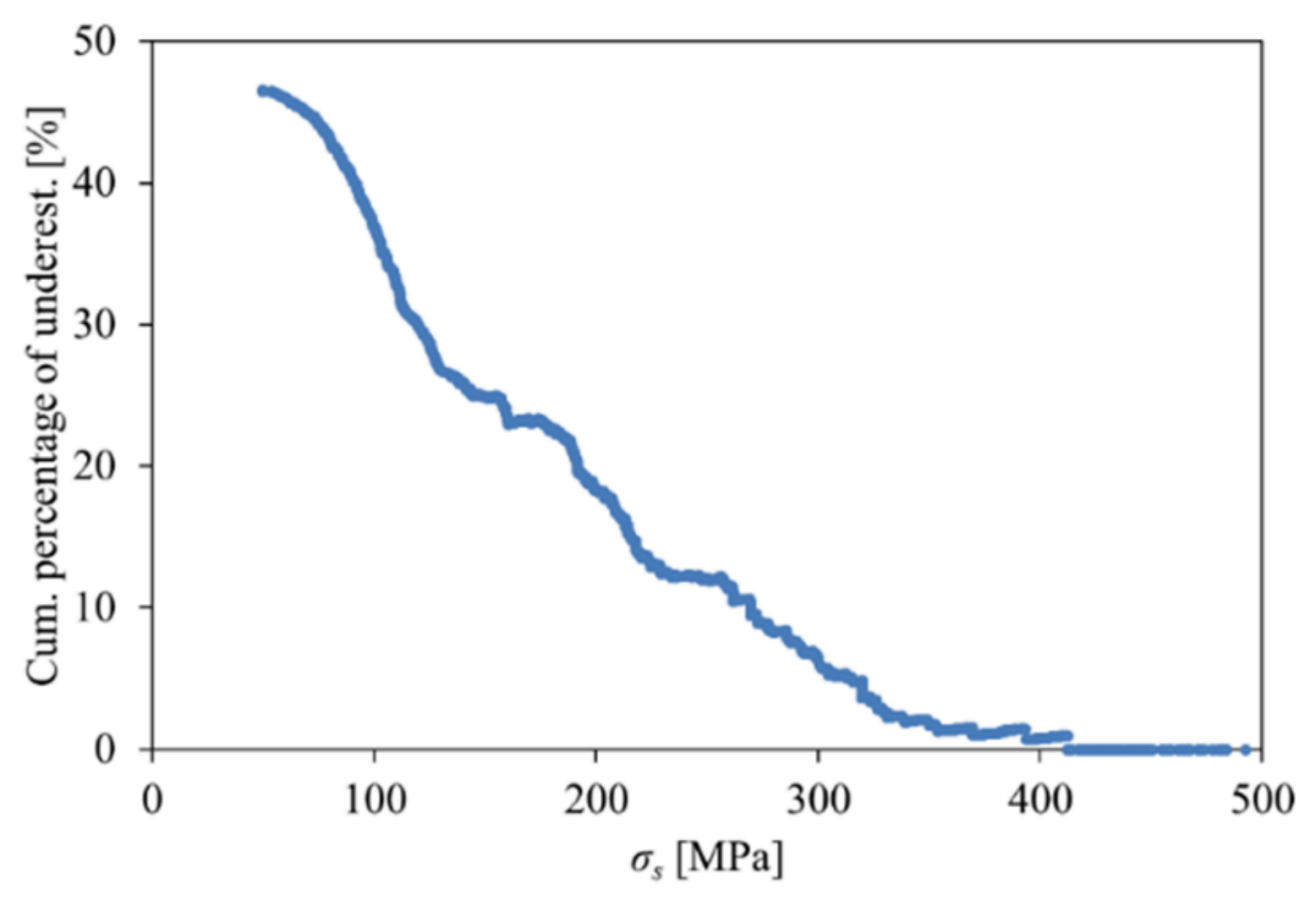
Cumulative percentage of EC2 underestimation vs. reinforcing-steel stress *σ_s_*.

**Figure 7:**
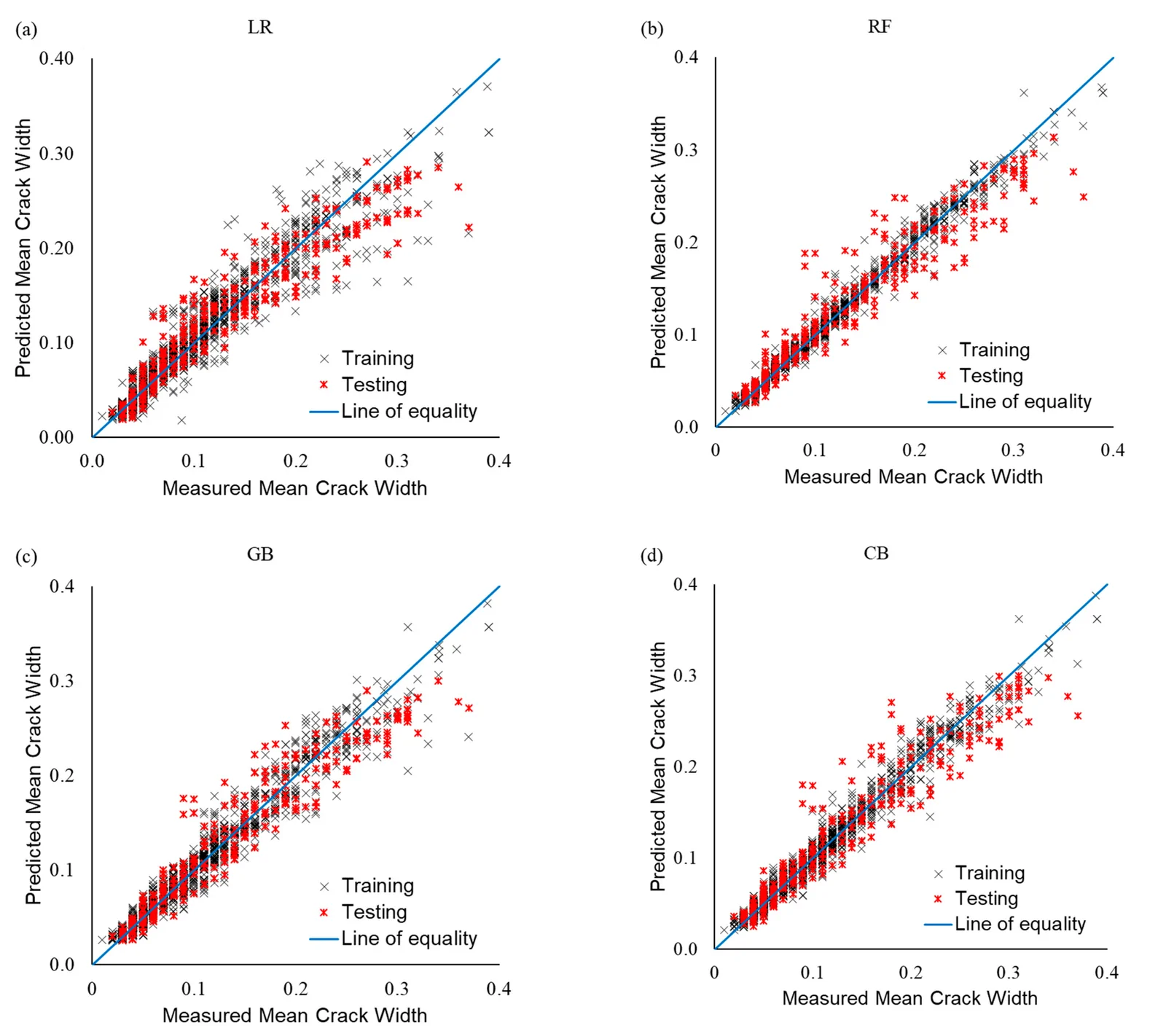
Comparison between experimentally measured and ML-predicted mean crack widths *w_m_* using imposed strain *ε_imp_* instead of reinforcement stress *σ_s_* as an input variable: (**a**) Linear Regression, (**b**) Random Forest, (**c**) Gradient Boosting, and (**d**) CatBoost.

**Figure 8:**
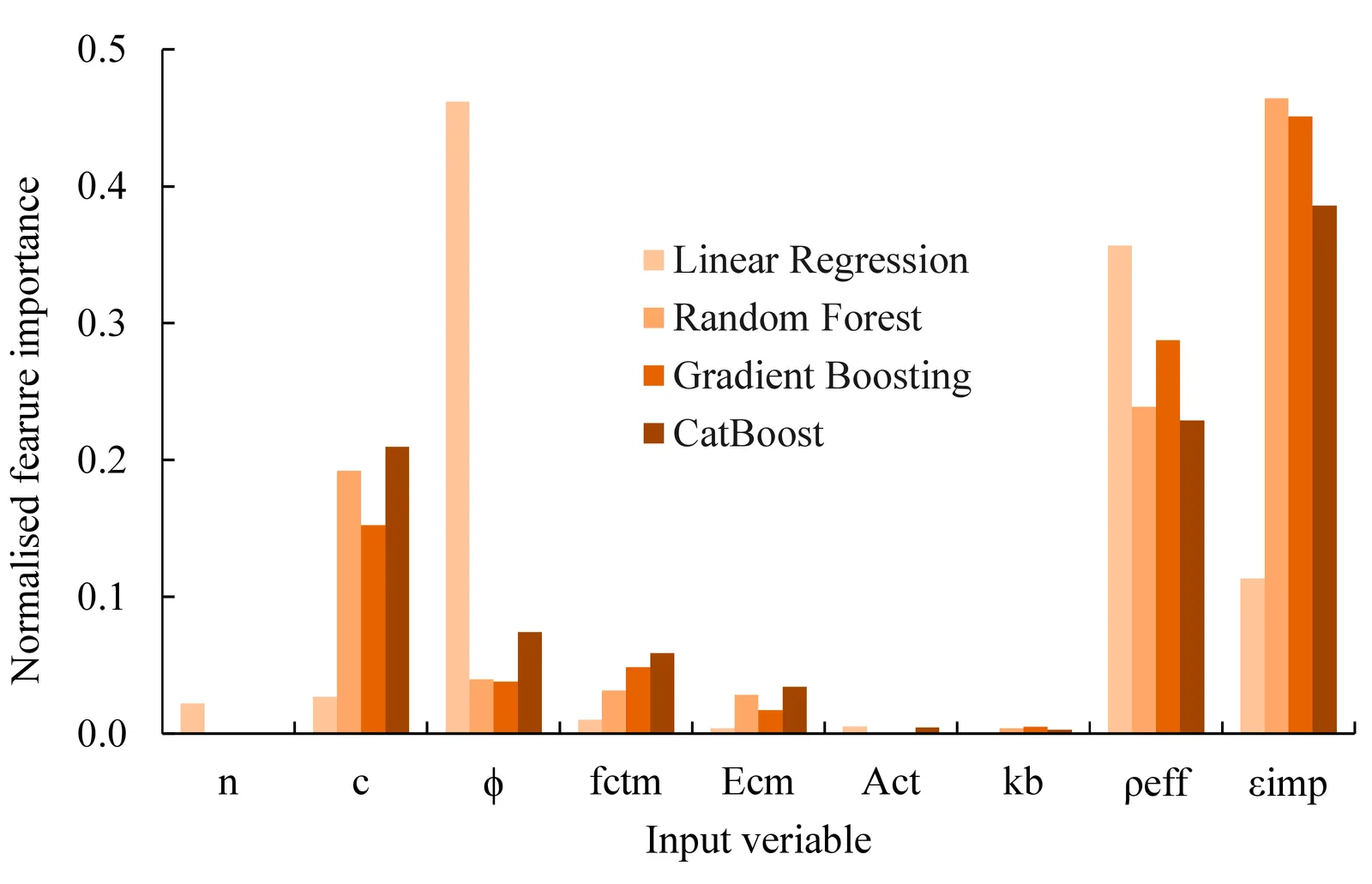
Normalised feature importance based on mean absolute SHAP values for ML models predicting the mean crack width *w_m_* using imposed strain *ε_imp_* instead of reinforcement stress *σ_s_* as an input variable.

**Figure 9:**
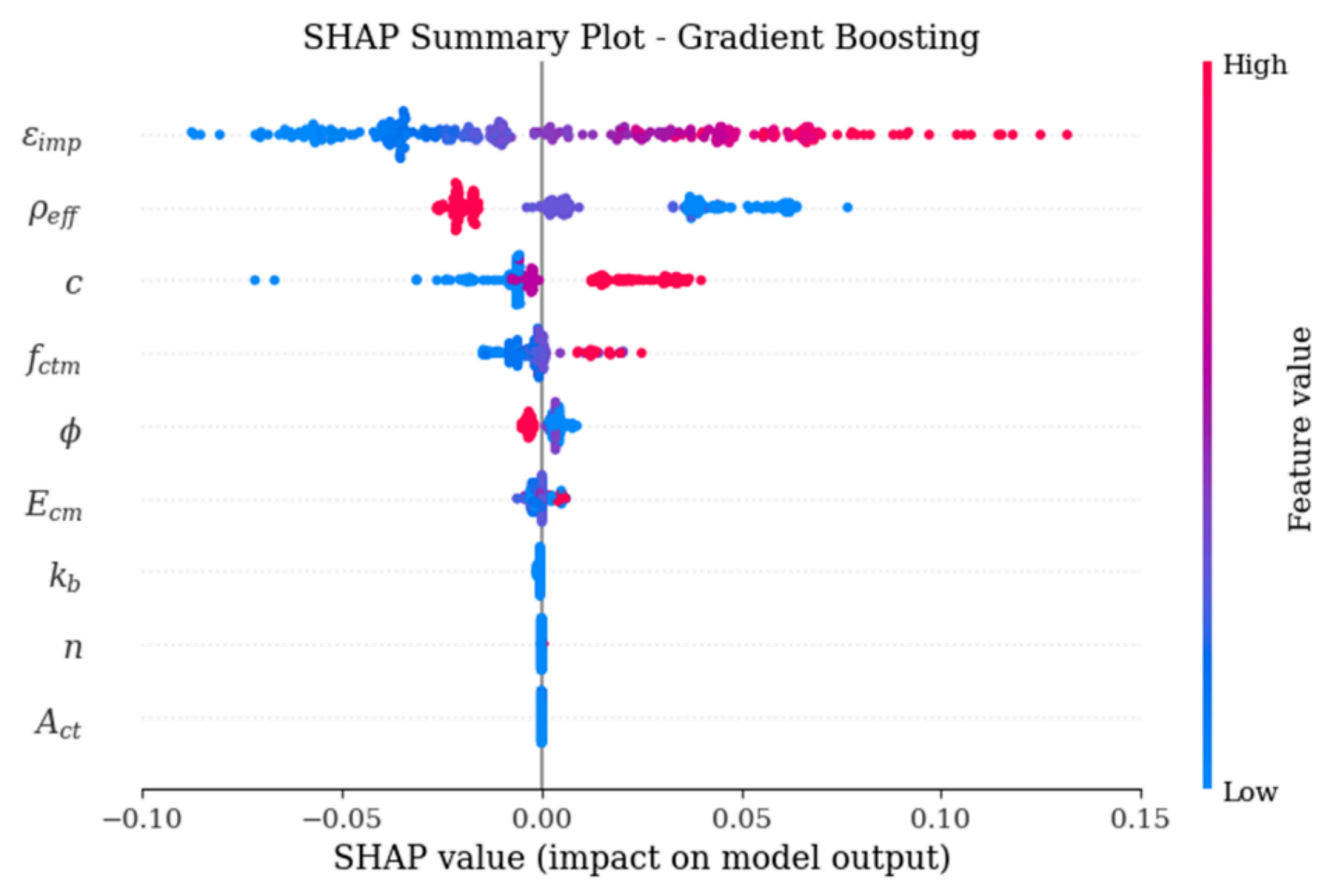
SHAP summary plot for the Gradient Boosting model predicting the mean crack width *w_m_*.

**Figure 10:**
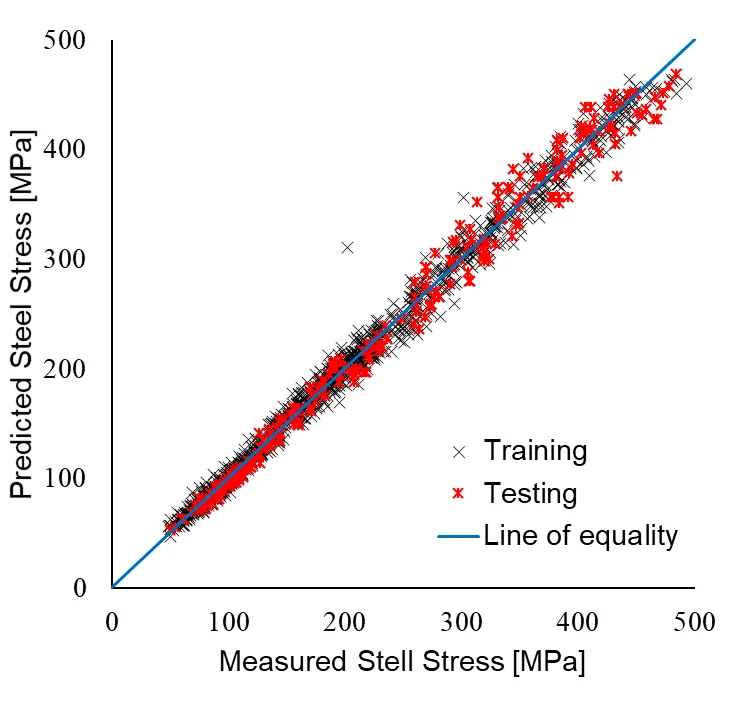
Comparison between measured reinforcement stresses and values predicted by the Gradient Boosting model.

**Figure 11:**
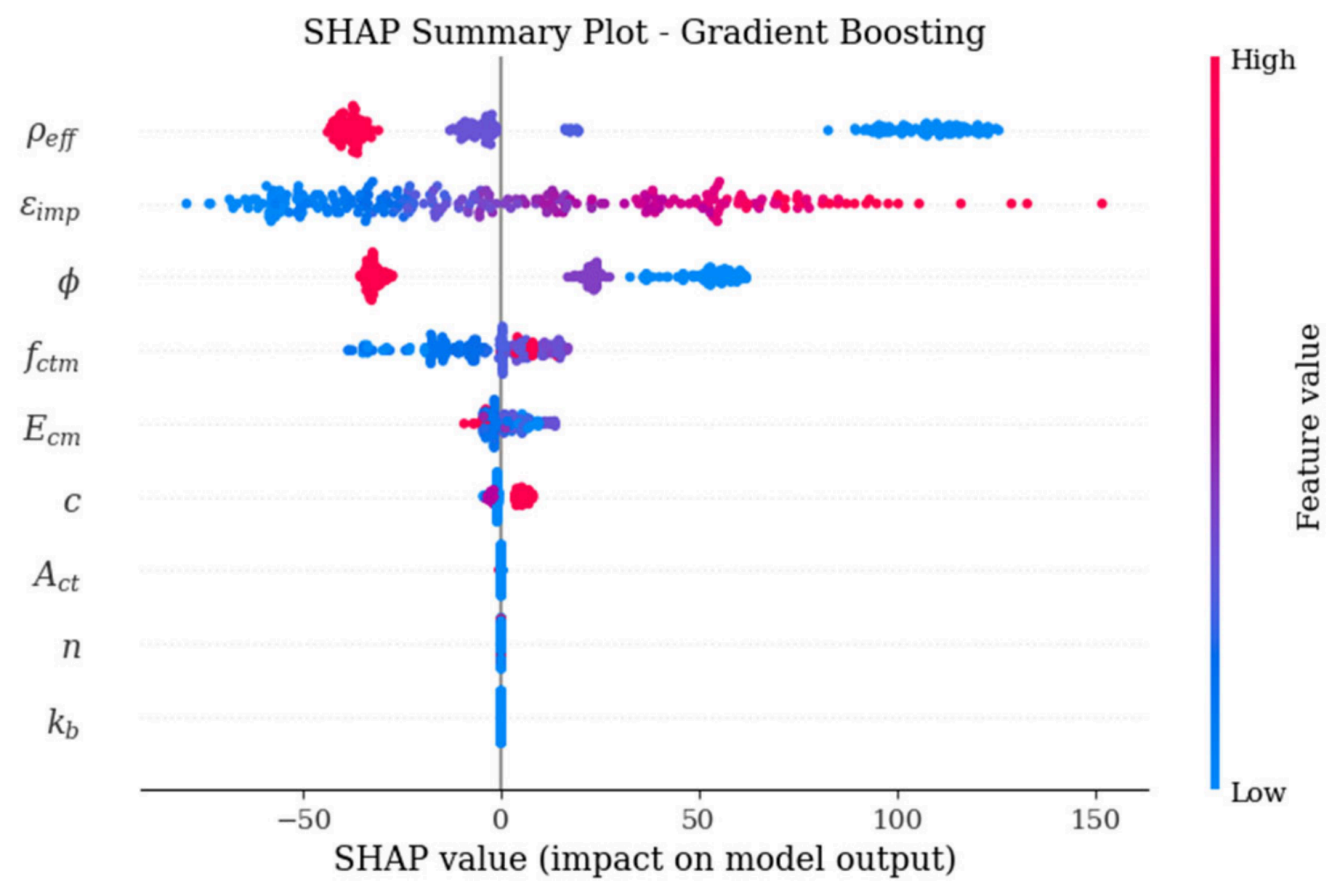
SHAP summary plot for the Gradient Boosting model predicting reinforcement stress *σ_s_* from imposed strain *ε_imp_*, material and geometrical input parameters.

**Figure 12:**
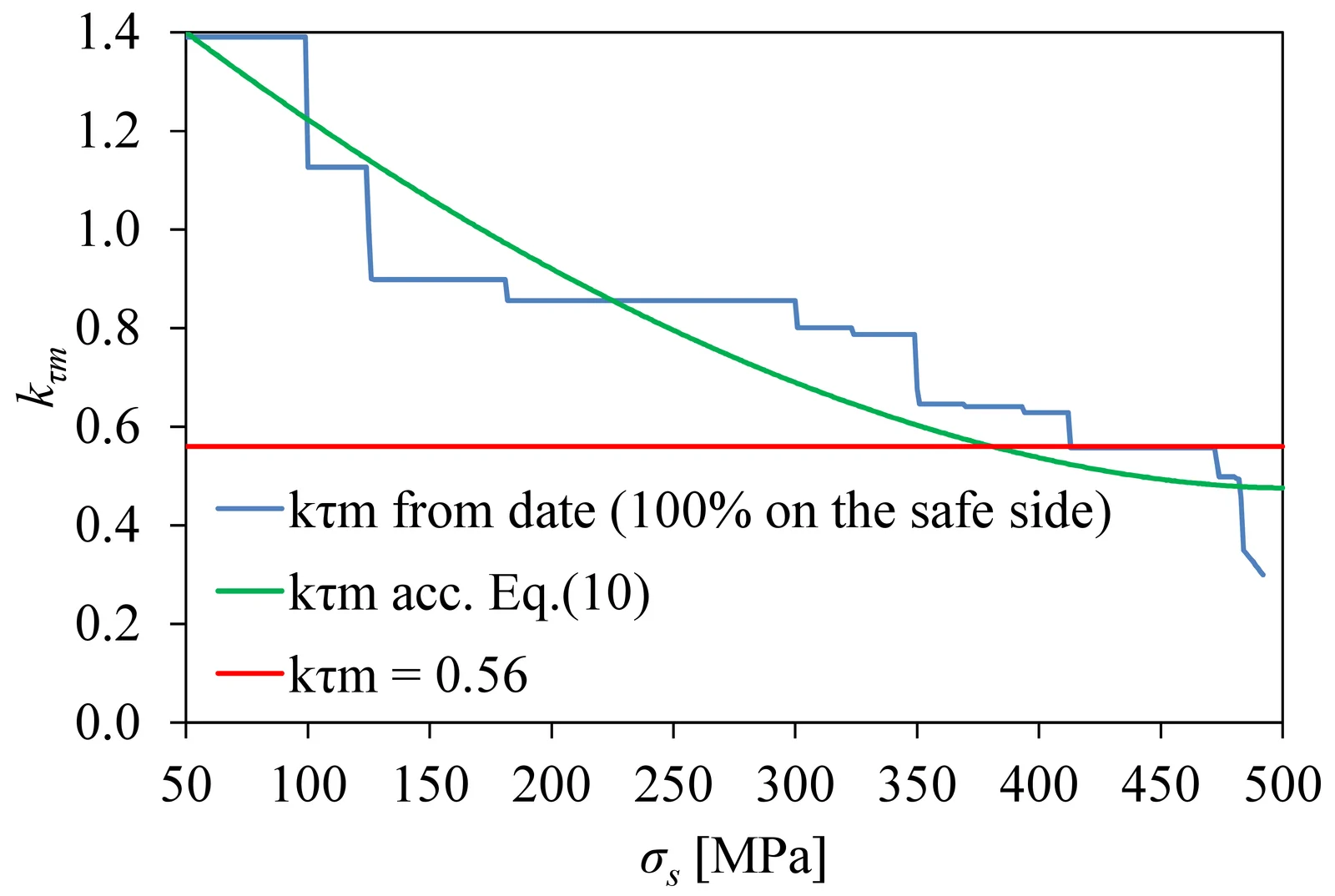
Dependence of the *k_τm_* coefficient on reinforcing-steel stress *σ_s_*.

**Figure 13:**
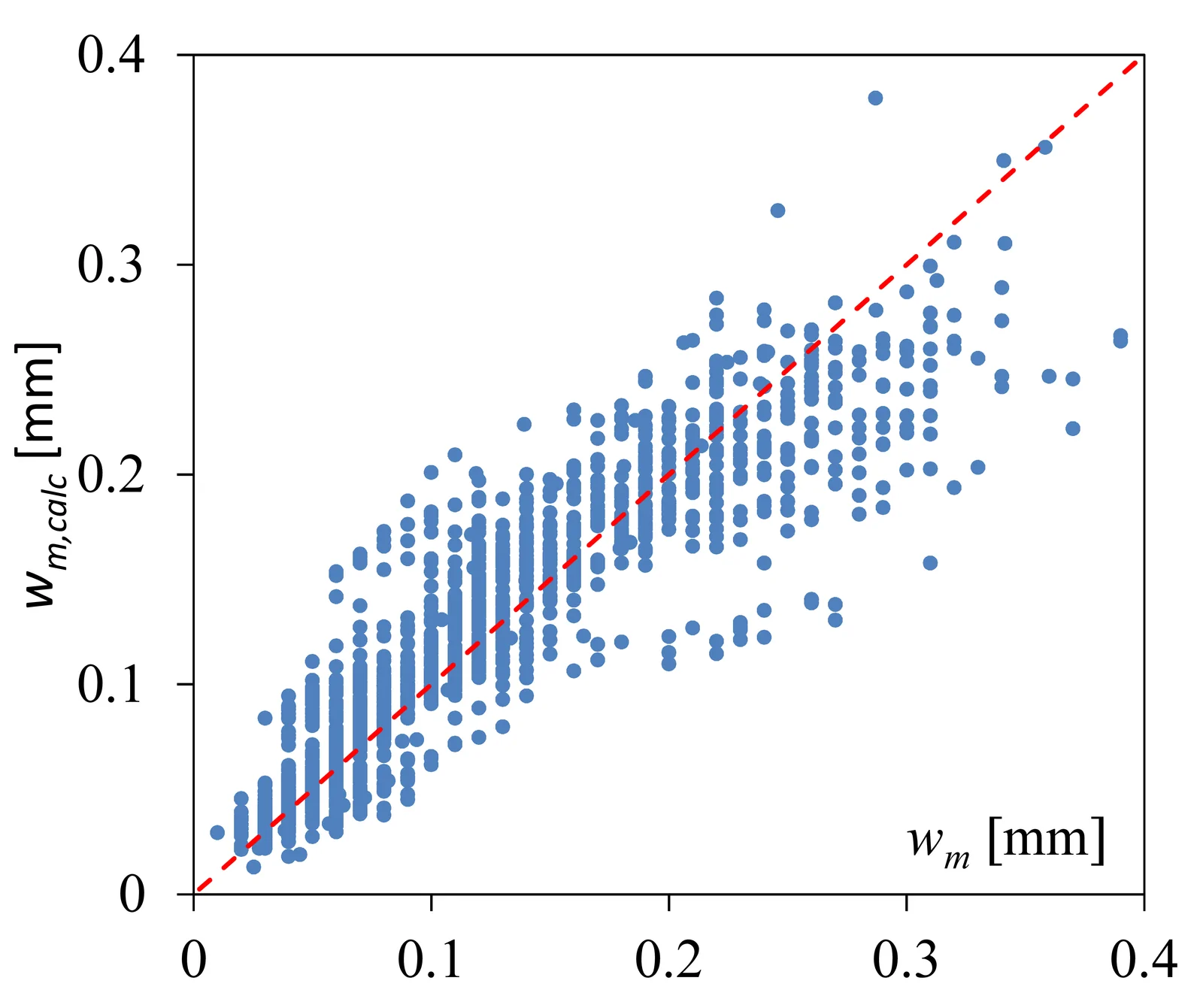
Scatter plot of experimentally obtained mean crack widths versus mean values calculated with the modified EC2023 model.

**Figure 14:**
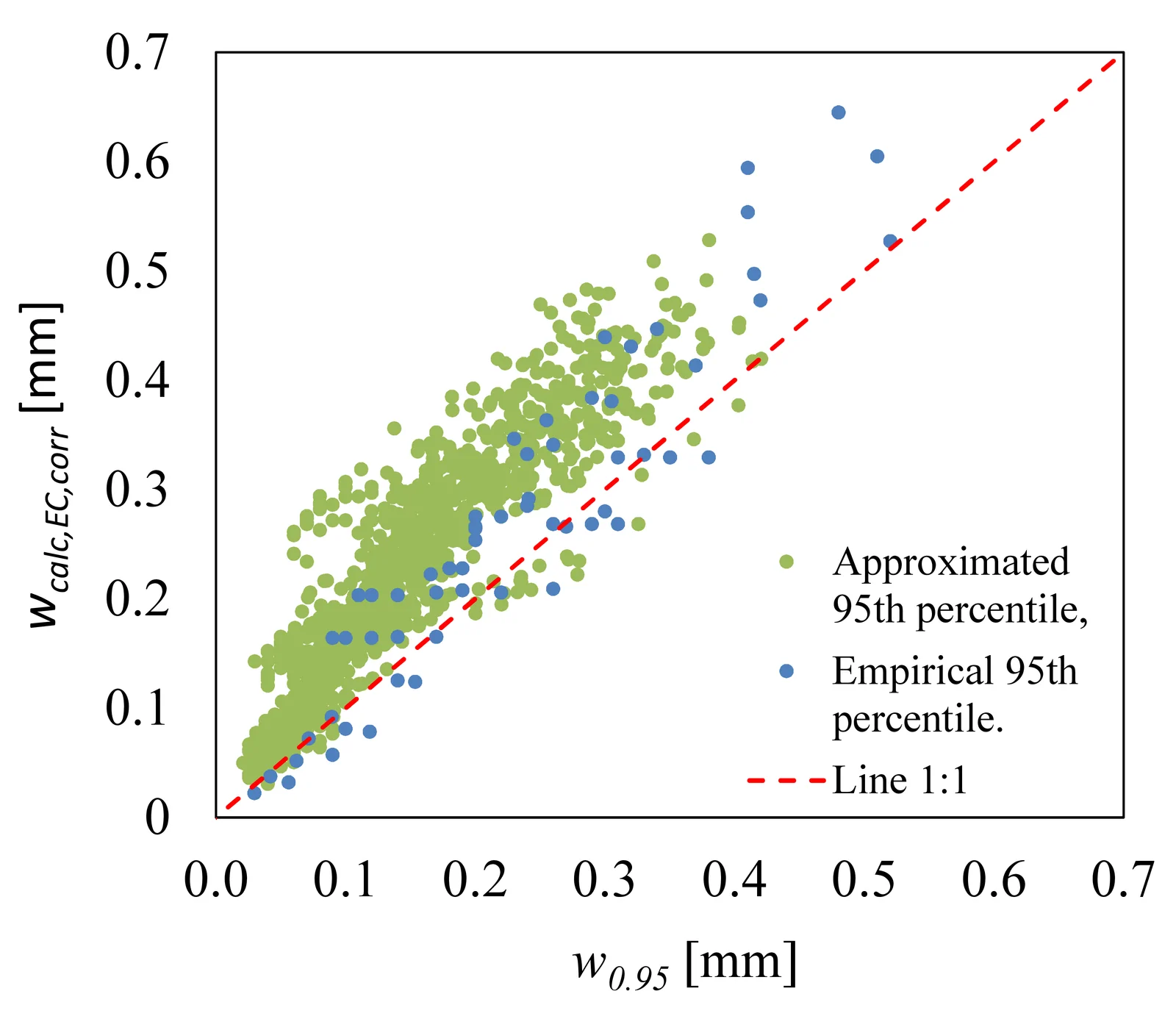
Comparison between the adopted 95th-percentile crack-width reference and crack widths calculated using the modified EC2023 model.

**Figure 15:**
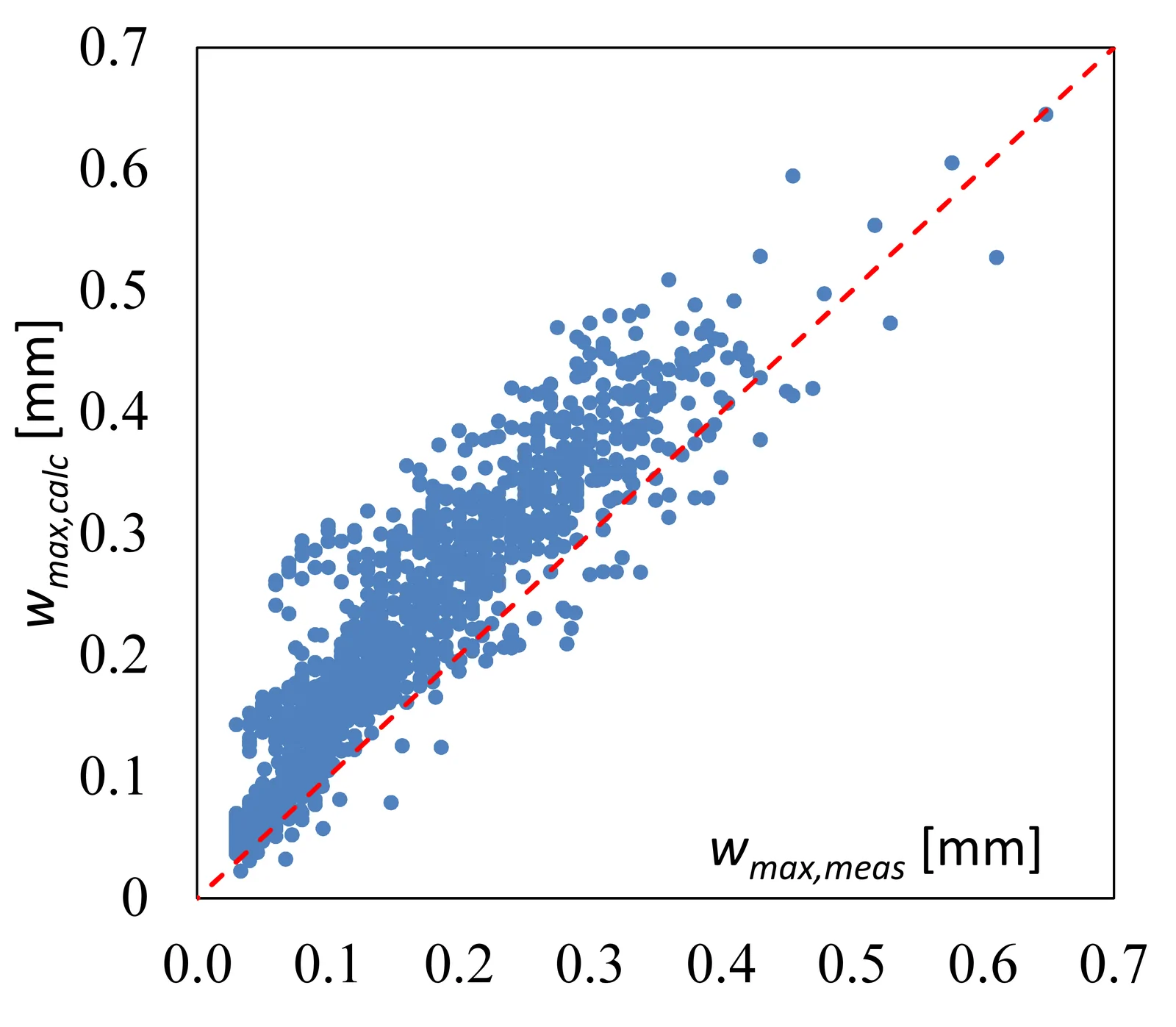
Scatter plots of the maximum measured crack widths versus the maximum values calculated with the modified EC2023 model. (The red dashed line represents the line of perfect agreement (1:1).)

**Figure 16:**
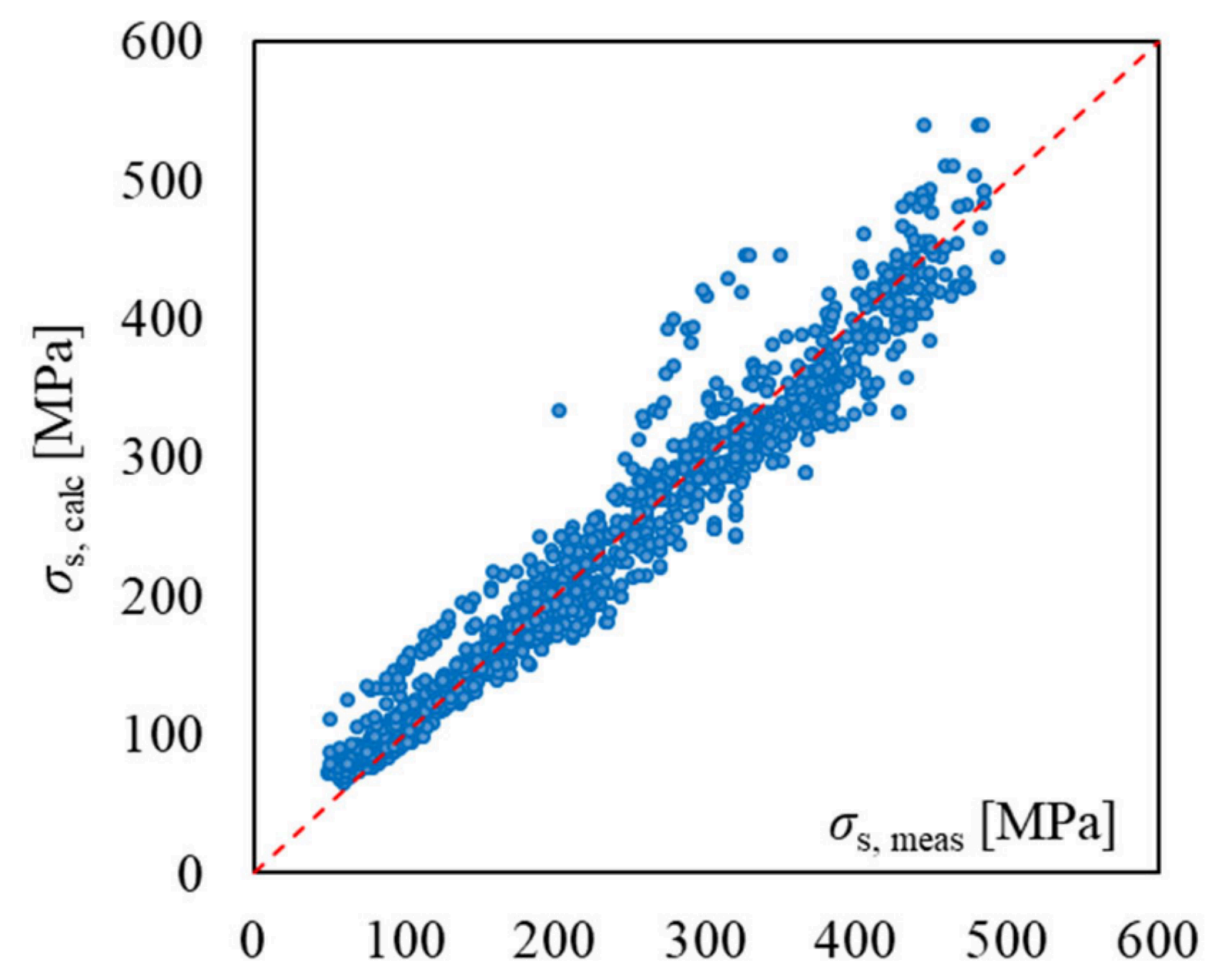
Comparison of reinforcement stresses calculated using the semi-empirical model with measured values for the calibration database (The red dashed line represents the line of perfect agreement (1:1)).

**Figure 17:**
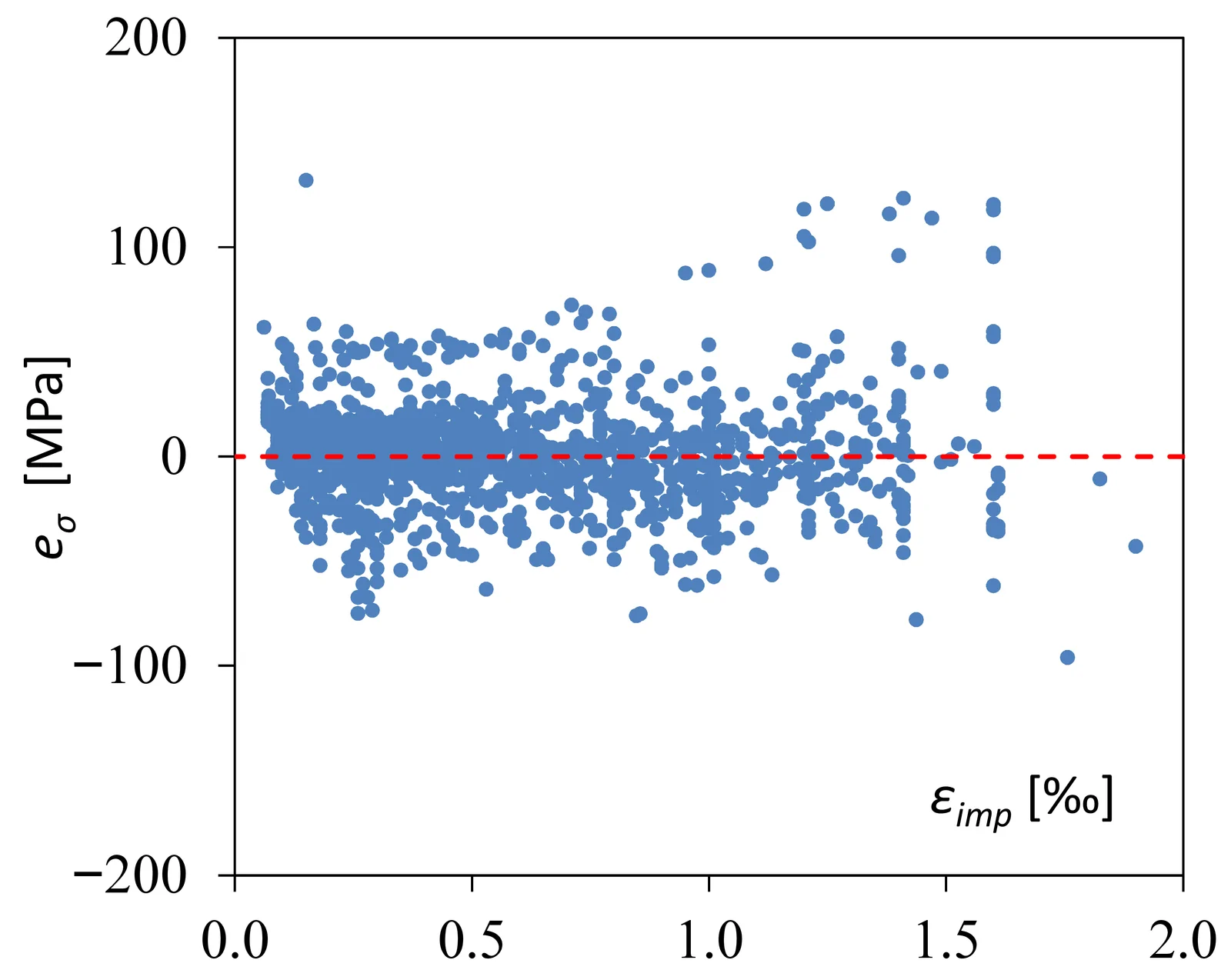
Residuals of the semi-empirical reinforcement-stress model plotted against the imposed strain (The red dashed line indicates zero concrete stress (σc = 0 MPa)).

**Figure 18:**
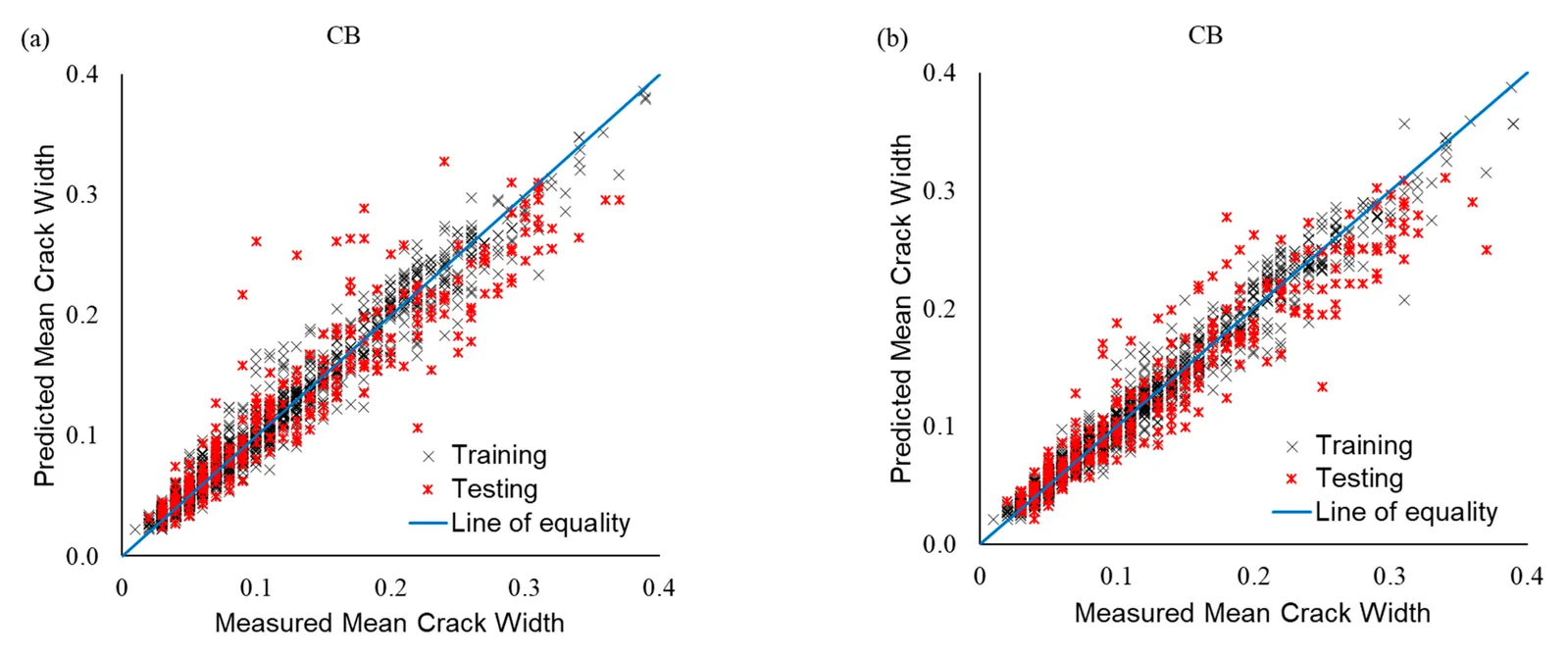
Comparison between experimentally measured and CatBoost-predicted mean crack widths (*w_m_*): (**a**) prediction based on measured reinforcement stress (*σ_s_*); (**b**) prediction based on ML-predicted reinforcement stress (*σ_s_*).

**Figure 19:**
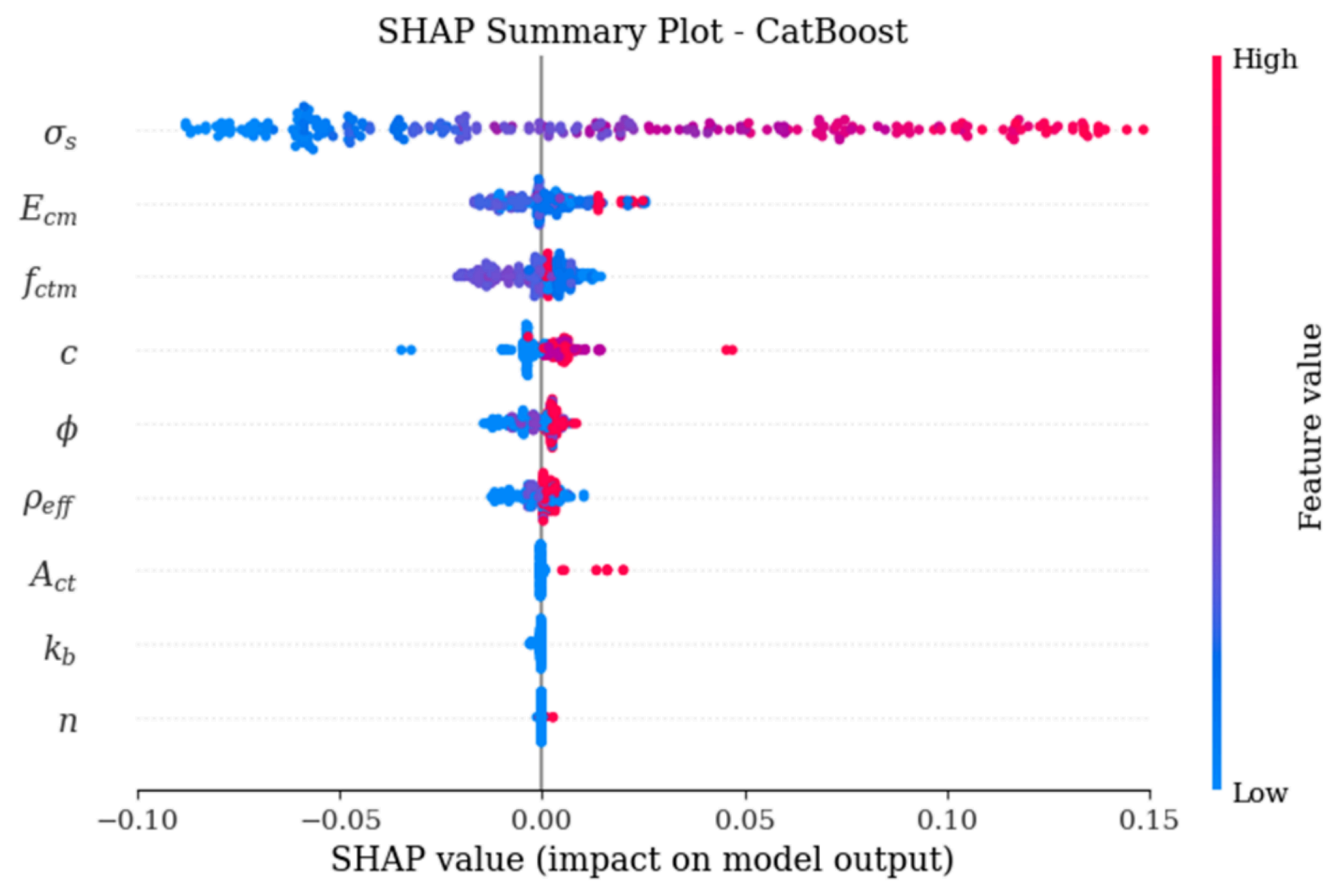
SHAP summary plot for the CatBoost model predicting the mean crack width *w_m_* using ML-predicted reinforcement stress *σ_s_* as the primary deformation-related input variable.

**Figure 20:**
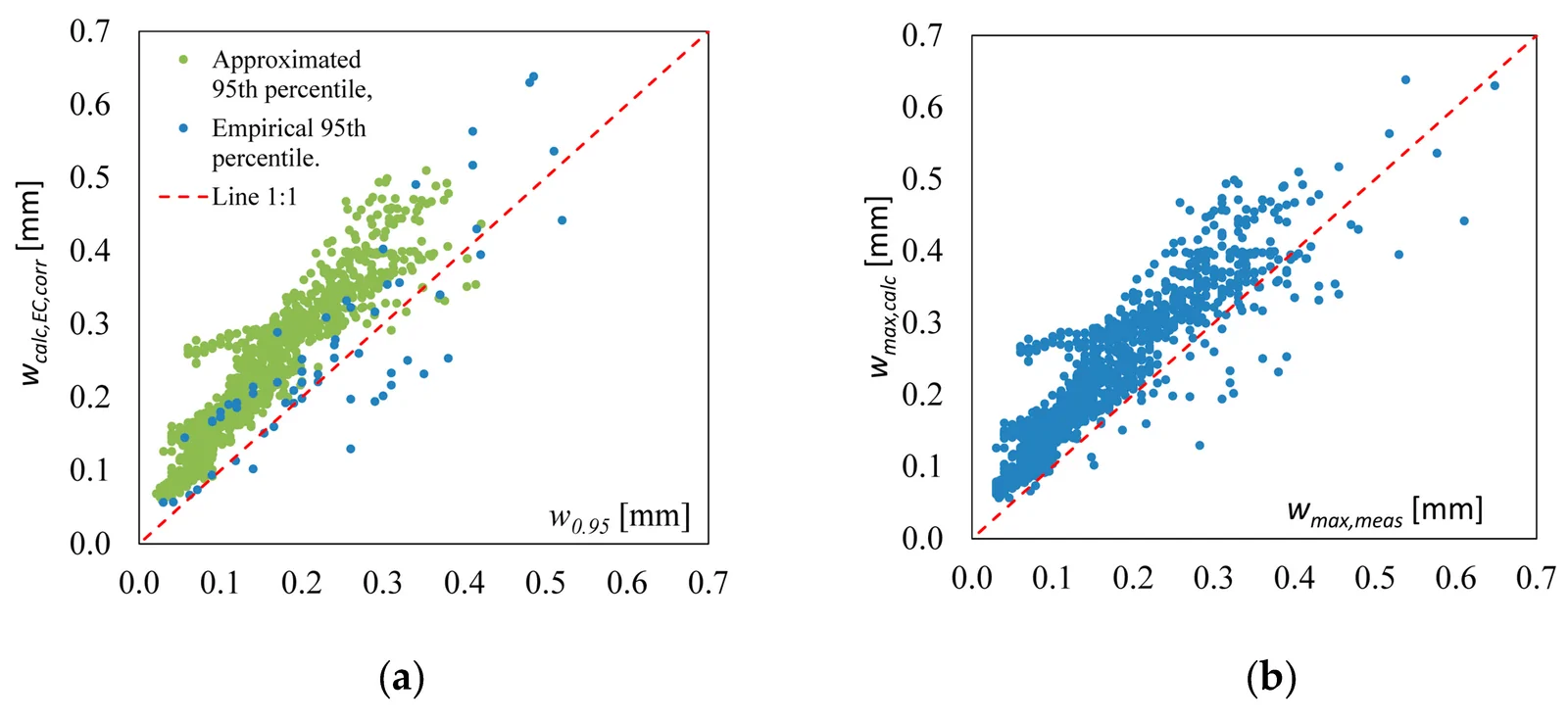
Verification of the modified EC2023 procedure with the *σ_s_*(*ε_imp_*) model: (**a**) comparison with *w*_0.95_; (**b**) comparison with *w_max_*.

**Table 1:** Sequential screening of the measurement-stage database.

Source Study	Retained Specimens	Initial Measurement-Stage Observations	Unloading (e.g., Decreasing-Stress Stages) or No Change in Stress Excluded	Observations with *w_max_* < 0.03 mm Excluded	Observations with *σ_s_* > 500 MPa Excluded	Final Retained Observations
Farra and Jaccoud [17]	121	3039	1180	431	15	1413
Rimkus and Gribniak [18]	10	20	0	0	0	20
García and Pérez Caldentey [19]	6	40	0	0	0	40
Total	137	3099	1180	431	15	1473

Note: The exclusion criteria were applied from left to right. Each observation was counted only once, under the first applicable criterion.

**Table 2:** Statistical characteristics of the filtered measurement-level experimental database used for code-based assessment and machine-learning analyses.

Data	Symbol	Unit	Min	Max	Mean	SD	Median
Concrete cover	*c*	mm	15.0	82.0	42.25	5.34	43.0
Bar diameter	*ϕ*	mm	10.0	25.0	15.97	4.09	14.0
Mean tensile strength of concrete	*f_ctm_*	Mpa	2.13	4.69	2.86	0.47	2.81
Mean modulus of elasticity of concrete	*E_cm_*	Gpa	27.00	41.80	31.66	3.80	30.30
Concrete area in tension	*A_ct_*	m^2^	0.0100	0.1575	0.0142	0.0240	0.010
Bond coefficient	*k_b_*	–	0.9	1.2	0.902	0.027	0.900
Reinforcement ratio	*ρ*	%	0.785	3.140	2.100	0.972	1.539
Imposed strain	*ε_imp_*	‰	0.061	1.900	0.565	0.390	0.470
Mean crack width	*w_m_*	mm	0.010	0.390	0.120	0.077	0.100
95th-percentile crack width	*w*_0.95_	mm	0.021	0.489	0.139	0.088	0.116
Maximum crack width	*w_max_*	mm	0.030	0.648	0.154	0.099	0.130
Reinforcement stress	*σ_s_*	Mpa	49.0	493.0	210.5	113.2	189.1

Note: The statistics are based on 1473 measurement-level observations extracted from successive imposed-strain stages. Therefore, the observations should not be interpreted as fully independent specimen-level test results.

**Table 3:** Pearson correlation coefficients between selected input parameters and the calculated crack width *w_k_*_,_*_cal_*_._

Parameter	Pearson Correlation with *w_k_*_,_*_cal_*
*σ_s_*	0.799
*h*	0.180
*A_s_*	0.159
*ϕ*	0.159
*c*	0.083
*f_ct_*_,_ *_eff_*	−0.063
*E_cm_*	−0.065

**Table 4:** Performance metrics of ML models for direct prediction of mean crack width *w_m_* using imposed strain *ε_imp_* and material, geometrical, and reinforcement-related input parameters

Model	Training			Testing		
	*R* ^2^	RMSE [mm]	MAE [mm]	*R* ^2^	RMSE [mm]	MAE [mm]
Linear Regression	0.901	0.023	0.016	0.887	0.031	0.022
Random Forest	0.984	0.009	0.006	0.904	0.027	0.018
Gradient Boosting	0.950	0.017	0.012	0.914	0.026	0.019
CatBoost	0.969	0.013	0.009	0.906	0.027	0.018

**Table 5:** Performance metrics of ML models for predicting reinforcement stress *σ_s_* from imposed strain *ε_imp_* and material, geometrical, and reinforcement-related input parameters.

Model	Training			Testing		
	*R* ^2^	RMSE [MPa]	MAE [MPa]	*R* ^2^	RMSE [MPa]	MAE [MPa]
Linear Regression	0.933	28.5	18.9	0.946	29.2	22.3
Random Forest	0.997	5.5	3.9	0.982	16.7	12.0
Gradient Boosting	0.991	10.3	7.3	0.987	13.9	10.2
CatBoost	0.995	7.9	5.9	0.985	15.1	10.8

**Table 6:** Performance metrics of the CatBoost model for prediction of mean crack width (*w_m_*) using measured and ML-predicted reinforcement stress (*σ_s_*).

Model	Training			Testing		
	*R* ^2^	RMSE [mm]	MAE [mm]	*R* ^2^	RMSE [mm]	MAE [mm]
Measured (*σ_s_*)	0.958	0.015	0.011	0.851	0.033	0.022
ML-predicted (*σ_s_*)	0.965	0.014	0.01	0.891	0.029	0.02

**Table 7:** Summary of the performance of the investigated crack-width prediction approaches.

Approach	Treatment of *σ_s_*	Key Outcome
Original EC2023 model	*σ_s_* required as input	Mean *w_m_*: *R*^2^ = 0.72, MAE = 0.033 mm, RMSE = 0.041 mm; 89% underestimation. For *w*_0.95_: approx. 47% underestimation.
MC2020 model	*σ_s_* required as input	Mean *w_m_*: *R*^2^ = 0.70, MAE = 0.033 mm, RMSE = 0.042 mm; 89.5% underestimation. For *w*_0.95_: 26.2% underestimation.
Modified EC2023 model	Measured *σ_s_* used	Mean *w_m_*: *R*^2^ = 0.83, MAE = 0.022 mm, RMSE = 0.032 mm. Underestimation: 3.4% for *w*_0.95_ and 5.5% for *w_max_*.
Complete modified EC2023 procedure	*σ_s_* calculated from *σ_s_*(*ε_imp_*)	*w_calc_* ≥ *w*_0.95_ in 98.24% of observations. Underestimation: 3.93% for *w_max_*; mean underestimation = 0.047 mm.
Direct *ε_imp_*-based CatBoost model	*σ_s_* excluded	Testing prediction of *w_m_*: *R*^2^ = 0.906, RMSE = 0.027 mm, MAE = 0.018 mm.
CatBoost model with ML-predicted *σ_s_*	*σ_s_* predicted by ML stress model	Testing prediction of *w_m_*: *R*^2^ = 0.891, RMSE = 0.029 mm, MAE = 0.02 mm.

Note: Underestimation denotes calculated crack widths below the corresponding reference value: measured mean crack width (*w_m_*), estimated upper-tail crack width (*w*_0.95_), or maximum measured crack width (*w_max_*).

## Data Availability

The experimental data used for model assessment and calibration were derived from Farra and Jaccoud [17] (https://infoscience.epfl.ch/entities/publication/6b8d08b2-4e4d-4b63-9185-0ac202003ae6, accessed on 22 September 2026), Rimkus and Gribniak [18] (https://doi.org/10.1016/j.conbuildmat.2017.05.029), and García and Pérez Caldentey [19] (https://doi.org/10.1016/j.engstruct.2020.111134). Data published with these sources can be consulted through the respective publications and their associated materials, where available. Additional detailed experimental data from the programme described in [19] were kindly provided by Alejandro Pérez Caldentey for the present analysis. These additional data are subject to third-party restrictions and cannot be redistributed by the authors. The model equations and aggregate numerical results generated in this study are reported in the article.

## Supplementary Materials

The following supporting information can be downloaded at: https://www.mdpi.com/article/10.3390/ma19194132/s1, Section S1: Equation-by-equation implementation of the proposed calculation procedure.
