# Supplementary material for: Bond-Transfer-Controlled Crack-Width Modelling of Reinforced Concrete as a Cement-Based Composite Under Imposed Strains
Source: Materials (Basel). 2026 Sep 28;19(19):4132. doi: 10.3390/ma19194132 (PMC13665807; doi:10.3390/ma19194132)
Supplement: Supplementary file 1 [file materials-19-04132-s001.zip › materials-4568840-supplementary.pdf]

Supplementary material

For the manuscript: "Bond-transfer-controlled crack-width modelling of reinforced concrete as a cement-based composite under imposed strains"

Section S1. Equation-by-equation implementation of the proposed calculation procedure

This appendix presents the final calculation sequence of the proposed procedure. The procedure combines the modified EC2023 crack-width formulation with a semi-empirical relationship for estimating reinforcement stress under imposed strain. Consequently, the steel stress is not assumed independently, but is calculated from geometrical, material, reinforcement-related, and imposed-strain parameters. The notation is consistent with the manuscript.

S1. Reinforcement-stress model

In the first step, the reinforcement stress is calculated as a function of imposed strain, bar diameter, mean tensile strength of concrete and effective reinforcement ratio:

$$\sigma_{s,calc} = 0.806\frac{100f_{ctm}}{\rho_{eff}} + \varepsilon_{imp}\left(42.135 + 11.162\rho_{eff} + 6.043\frac{\phi}{\rho_{eff}}\right) + 7.104\varepsilon_{imp}^2 \qquad \text{(S.1)}$$

In Eq. (A.1), the following units must be used: $\sigma_{s,calc}$ in MPa, ϕ in mm, f_{ctm} in MPa, ρ_{eff} in %, and ε_{imp} in ‰.

S2. Modified crack-width model

The crack width is calculated using the general EC2023 format:

$$w_{calc} = k_w k_{1/r}\, s_{r,\mathrm{m},mod}(\varepsilon_{sm} - \varepsilon_{cm})_{mod} \qquad \text{(S.2)}$$

In Eq. (A.2), k_w = 1.0 may be used for comparison with measured mean crack widths, whereas k_w = 1.7 may be used for design-oriented or upper-tail crack-width prediction, $k_{1/r}$ = 1.0.

The modification of the crack-spacing term is introduced through the following stress-dependent bond coefficient:

$$k_{1,mod} = 0.56\left[0.85 + 1.65\left(\frac{500-\sigma_s}{450}\right)^{1.805}\right] \qquad \text{(S.3)}$$

In Eq. (A.3), the reinforcement stress σ_s is substituted in MPa. The value of $k_{1,mod}$ increases at low reinforcement stress levels, which corresponds to the single-crack or non-stabilised cracking range, and decreases as the stress increases and the cracking pattern becomes more developed.

The crack-spacing contribution:

$$\frac{1}{7.2}\frac{\phi}{\rho_{eff}} \qquad \text{(S.4)}$$

is replaced by:

$$k_{1,mod}k_4\frac{\phi}{\rho_{eff}} \qquad \text{(S.5)}$$

where:

$$k_4 = 0.25 \qquad \text{(S.6)}$$

This preserves the structure of the EC2023 formulation while introducing a variable level of effective bond stress between concrete and reinforcement.

The second modification concerns the mean strain-difference term. The constant coefficient k_t is replaced by an effective coefficient dependent on the cracking stage:

$$k_{t,eq} = 0.43 + 0.15\frac{\sigma_{cr}}{\sigma_s} \tag{S.7}$$

The ratio σ_{cr}/σ_s is used as a practical indicator of crack-pattern development. Accordingly, $k_{t,eq}$ decreases with increasing reinforcement stress and with the progressive formation of the crack pattern.

The modified difference between the mean reinforcement and concrete strains is then calculated as:

$$(\varepsilon_{sm} - \varepsilon_{cm})_{mod} = \frac{\sigma_s - k_{t,eq}\frac{f_{ct,eff}}{\rho_{eff}}(1+\alpha_e\rho_{eff})}{E_s} \tag{S.8}$$

with the lower bound consistent with the EC-type formulation:

$$(\varepsilon_{sm} - \varepsilon_{cm})_{mod} \geq \left(1 - k_{t,eq}\right)\frac{\sigma_s}{E_s} \tag{S.9}$$

S3. Final calculation sequence

In the final procedure, the reinforcement stress σ_s used in Eqs. (A.3), (A.7), (A.8), and (A.9) is not an independently prescribed or measured quantity. It is calculated from Eq. (A.1):

$$\sigma_s = \sigma_{s,calc}\left(\phi, f_{ctm}, \rho_{eff}, \varepsilon_{imp}\right) \tag{S.10}$$

The final model can therefore be expressed in the compact form given in Eq. (A.2).

The above formulations directly relate the known or assumed imposed strain (ε_{imp}) to the calculated crack width. In contrast to the standard EC2023 procedure, the reinforcement stress is no longer an undefined input for members restrained at opposite ends, but is obtained from the semi-empirical stress-estimation relationship.

S4. Calibration domain and limitations

The proposed procedure should be applied within the range of parameters used for calibration:

$$10 \leq \phi \leq 25 \text{ mm} \tag{S.11}$$

$$2.13 \leq f_{ctm} \leq 4.69 \text{ MPa} \tag{S.12}$$

$$0.785 \leq \rho_{eff} \leq 3.6 \text{ \%} \tag{S.13}$$

$$0.061 \leq \varepsilon_{imp} \leq 1.9 \text{ ‰} \tag{S.14}$$

which corresponds approximately to the following reinforcement-stress range

$$49 \leq \sigma_s \leq 493 \text{ MPa} \tag{S.15}$$

The proposed relationship is semi-empirical and was identified for the analysed database of RC members subjected to imposed strains. It should not be interpreted as a universal code equation. Rather, it is a regression-based calculation procedure with partial mechanical justification, intended for the members represented by the calibration database. Extrapolation beyond the above ranges may lead to increased uncertainty.
